# Intensifying Drought Patterns and Agricultural Water Stress in Erbil Governorate, Iraq: A Spatiotemporal Climate Analysis

**DOI:** 10.1002/gch2.202500491

**Published:** 2025-12-08

**Authors:** Karrar Mahdi, Heman Abdulkhaleq A. Gaznayee, Payman Hussein Aliehsan, Sara H. Zaki, Dawod R. Keya, Kawa Hakzi, Fuad Alqrinawi, Michel Riksen

**Affiliations:** ^1^ Soil Physics and Land Management Group Wageningen University & Research Wageningen Netherlands; ^2^ Department of Forestry College of Agriculture Engineering Science Salahaddin University‐Erbil Erbil Iraq; ^3^ TISHK Center for Kurdistan Studies Cologne Germany; ^4^ Soil and Water Department College of Agricultural Engineering Sciences Salahaddin University‐Erbil Erbil Iraq; ^5^ Cultivision LTD Erbil Iraq; ^6^ School of Geography Earth and Environmental Sciences University of Birmingham Edgbaston UK; ^7^ Scientific Research Center Climate Change Unit‐ Salahaddin University‐Erbil Iraq

**Keywords:** agricultural water management, climate indices, drought patterns, evapotranspiration, SPI, spatiotemporal analysis

## Abstract

Drought is a prolonged lack of rainfall that causes water shortages for agricultural land by reducing soil moisture and limiting crop yields. This study assesses the impacts of drought in Erbil, Iraq, using 28 years of data from the southern, central, and northern regions. A soil‐water‐balance framework integrates precipitation with reference evapotranspiration (ETo), crop evapotranspiration (ETc), and actual evapotranspiration measures to address seasonal water deficits. According to the Standardized Precipitation Index (SPI), the area experienced basin‐wide droughts in 1998–2000, 2007–2009, a renewed event in 2020–2021, and significant negative anomalies in 2024–2025. Analyzing drought frequency shows that “near‐normal” conditions are most common (around 57%–79%), with occasional moderate to extreme events. The study reveals that winter wheat undergoes severe stress, with ETc reaching 200–250 mm month during dry years and ETa dropping to 15–30 mm/month, resulting in June deficits of nearly 277 mm. It also notes high variation in annual precipitation, with coefficients of variation (CV) ranging from 26.1% to 51.7%. ARIMA (1,0,1) models suggest weak persistence and zone‐specific accuracy, with MAPE values of 37.8% in the south, 33.9% in the central region, and 28.8% in the north. The results underscore the importance of climate‐resilient water and agricultural planning.

## 1 Introduction

Drought is one of the most significant environmental hazards worldwide, presenting serious challenges to water resources, food security, and socio‐economic stability [1]. Drought can therefore be viewed as a complex natural disaster that is difficult to identify (including its origin, duration, intensity, and extent), predict, and control within this broader context [2]. Droughts usually lead to water shortages caused by low rainfall, excessive water use, high evapotranspiration rates, or a mix of these factors [3]. Arid and semi‐arid regions are particularly vulnerable to droughts, with Iraq experiencing significant pressure on its water supplies and agricultural output [4]. Within Iraq, the Erbil Governorate is of particular interest due to its geographic position, economic importance, and ongoing challenges in balancing water demand and supply under intensifying climate pressures [5].

Agriculture in the Iraqi Kurdistan Region (KRI) suffered significant damage during times of drought, and vulnerable residents were seriously affected as well [6, 7]. Although there are sufficient water resources in the northern Iraq region, their availability remains limited and unpredictable, both spatially and temporally [8]. In essence, drought is the shortfall between precipitation and terrestrial water storage, which includes surface waters, underground aquifers, and water demand. This imbalance negatively affects agricultural productivity, environmental quality, and the overall economy [9, 10]. As per the National Oceanic and Atmospheric Administration (NOAA), Iraq has experienced severe droughts over the past decade due to reduced precipitation, high temperatures, decreased upstream water levels, and inefficient water usage.

Climate change has also caused a decrease in annual precipitation averages. All these factors have negatively impacted water resources and led to water scarcity [11, 12, 13]. While lack of typical precipitation remains a primary factor in causing such calamities, human activities also contribute [14, 15]. Recent studies have underscored the complex interplay between meteorological factors such as precipitation deficits and temperature anomalies and agricultural activities in arid environments [16]. Numerous studies have employed meteorological drought indicators to assess, track, and make decisions about such events. A standard tool used for characterizing drought is the Standardized Precipitation Index (SPI) [17]. It is a useful, uncomplicated indicator that can be evaluated across various temporal intervals when monitoring meteorological droughts. This characteristic of the methodology facilitates the comparison of drought occurrences across different locations and scales [8, 18].

The Soil Water Balance is a key concept in hydrology and agriculture that tracks water movement in the soil system monthly. It includes components like precipitation, evapotranspiration, crop water demand, groundwater recharge, drainage, and soil water content [19, 20]. Frequent droughts can cause significant winter wheat losses. Especially when drought and heat extremes coincide, the resultant impact would be even more harmful to human society and the natural environment [21, 22]. To mitigate this risk, establishing an index for monitoring and preventing drought damage is essential, along with understanding the specific spatiotemporal characteristics of winter wheat droughts [23]. Climate change and land‐use change mutually reinforce negative impacts and reduced resilience [24]. Understanding the relationship between evapotranspiration parameters in different drought scenarios is crucial for evaluating drought severity and its effects on water resources and crop yield [25]. Monitoring (ETo), (ETc), and (ETa) is vital for optimizing irrigation strategies, assessing drought stress on crops, and developing resilience measures [26].

Agricultural drought occurs when soil moisture is insufficient to support crop growth, leading to decreased productivity [27]. It emphasizes the effects of drought on agricultural systems, focusing on water availability in the root zone rather than total precipitation [20]. This form of drought can arise from a combination of meteorological drought (reduced precipitation) and increased evapotranspiration rates, resulting in critical soil water deficits for crops [30]. In addition, several studies have been conducted on spatiotemporal patterns of drought; however, most have focused primarily on detection techniques for droughts or analyzing the relationships between agricultural droughts and crop yields or precipitation averages using Landsat time‐series datasets [28]. Thus, there is a need for the thorough investigation of seasonal dynamics of droughts at the meteorological scale and vegetal spheres to determine spatiotemporal patterns of these phenomena [29]. Drought analysis requires considering several factors, including but not limited to: precipitation levels; soil moisture; potential evapotranspiration; vegetation condition; groundwater levels; surface water levels, etc. Correlation among different types of indices can be quite low because they do not necessarily anticipate similar trends, given their non‐linear relationship with each other [30, 31, 32].

Among the available statistical methods, the Autoregressive Integrated Moving Average (ARIMA) model has proven highly effective for analyzing and forecasting hydro‐climatic time series. ARIMA is widely recognized for its ability to capture stochastic processes, identify long‐term and short‐term trends, and generate reliable precipitation predictions [33, 34]. Its application in drought‐prone regions allows researchers and policymakers to assess precipitation variability, anticipate drought events, and develop adaptive management strategies. In particular, ARIMA‐based projections provide early warning signals, reduce uncertainty in water resource planning, and support decision‐making in agriculture amid increasing climatic stress. Therefore, precipitation forecasting is essential for sustainable water resource management, agricultural planning, and disaster risk reduction [35]. Historical precipitation data were analyzed using the ARIMA model, a well‐established time‐series forecasting technique [36], to identify spatiotemporal trends and predict future precipitation. Understanding these patterns is vital not only for agricultural planning but also for anticipating water scarcity, drought risk, and flood hazards. By applying ARIMA modeling across multiple meteorological stations in Erbil Governorate, this research aims to assess whether drought patterns are intensifying and how these changes translate into agricultural water stress [37]. This study investigates the spatiotemporal patterns of agricultural drought in Erbil Governorate, identifying reduced precipitation as the main cause. Using a 28‐year meteorological dataset (1998–2025) that includes SPI, ETo, ETc, ETa, CV, and precipitation, the research highlights distinct drought trends across the region's diverse topographies. The study's novelty is in combining multiple drought indices and exploring their relationships under semi‐arid conditions, providing insights into how these factors collectively impact agricultural productivity. What strategies can help mitigate these effects? Addressing this question supports SDG 2 (Zero Hunger) by showing how drought‐related soil moisture deficits threaten a farming‐dependent region, and SDG 13 (Climate Action) by demonstrating how climate change exacerbates drought through declining precipitation, increased evapotranspiration, and extreme weather events.

## 2 Materials and Methods

### 2.1 Study Area

**FIGURE 1:**
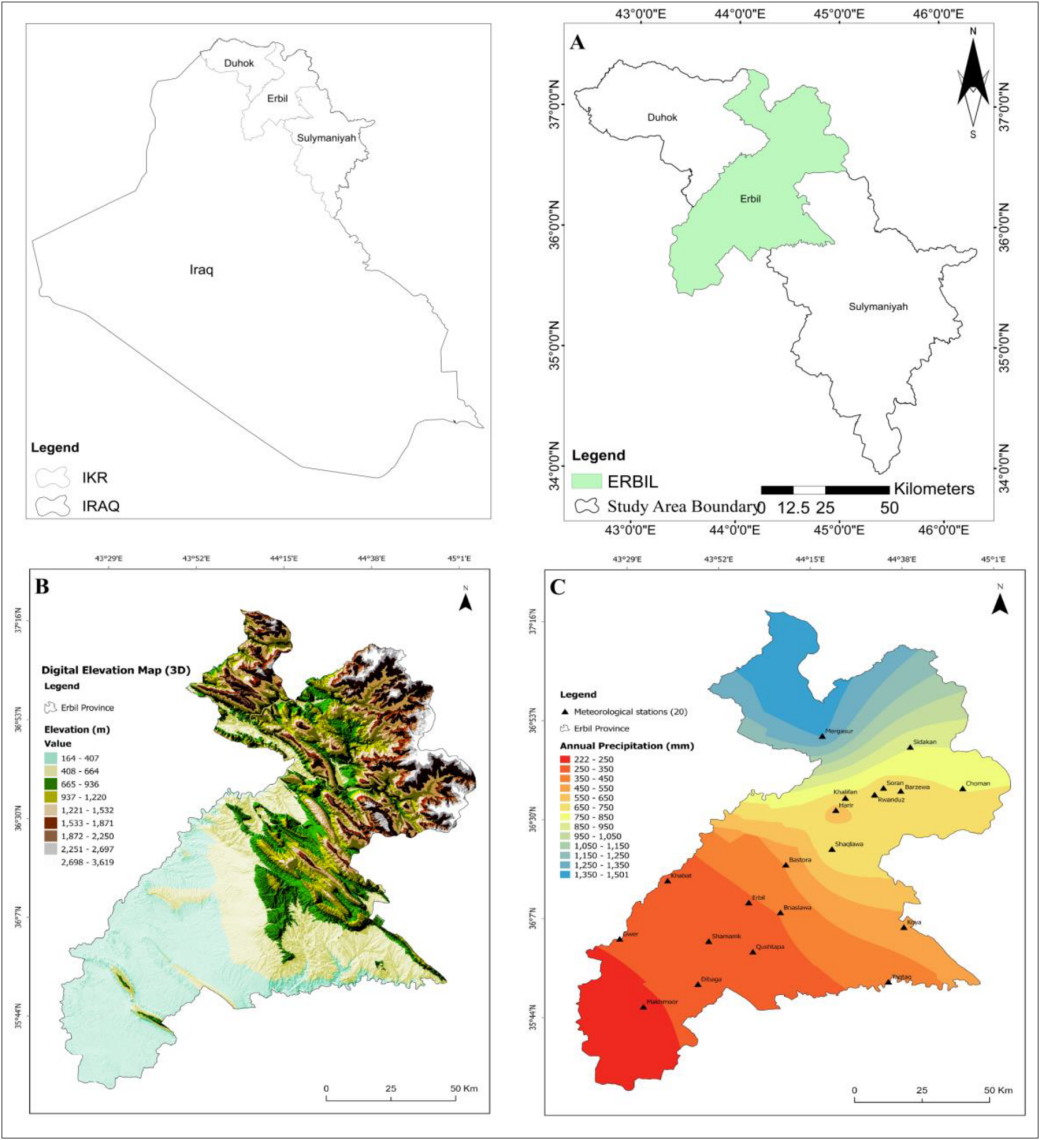
(A) Map depicting the geographical location of the research site. (B) Digital representation of the elevation model (DEM). (C) The spatial dispersion of yearly precipitation (mm/year) in Erbil from 1998 to 2021.

**FIGURE 2:**
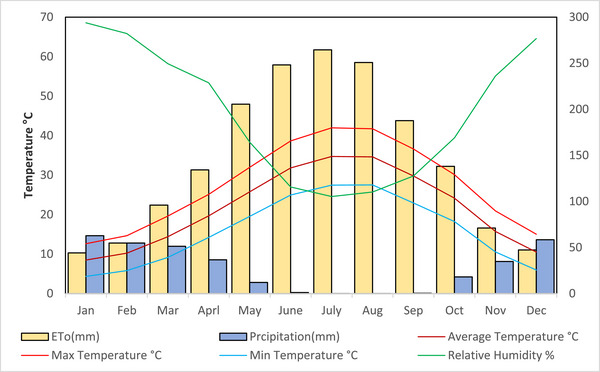
Monthly precipitation, relative humidity, potential evaporation, and the maximum, minimum, and average temperatures at Erbil are documented from 1998 to 2021.

**FIGURE 3:**
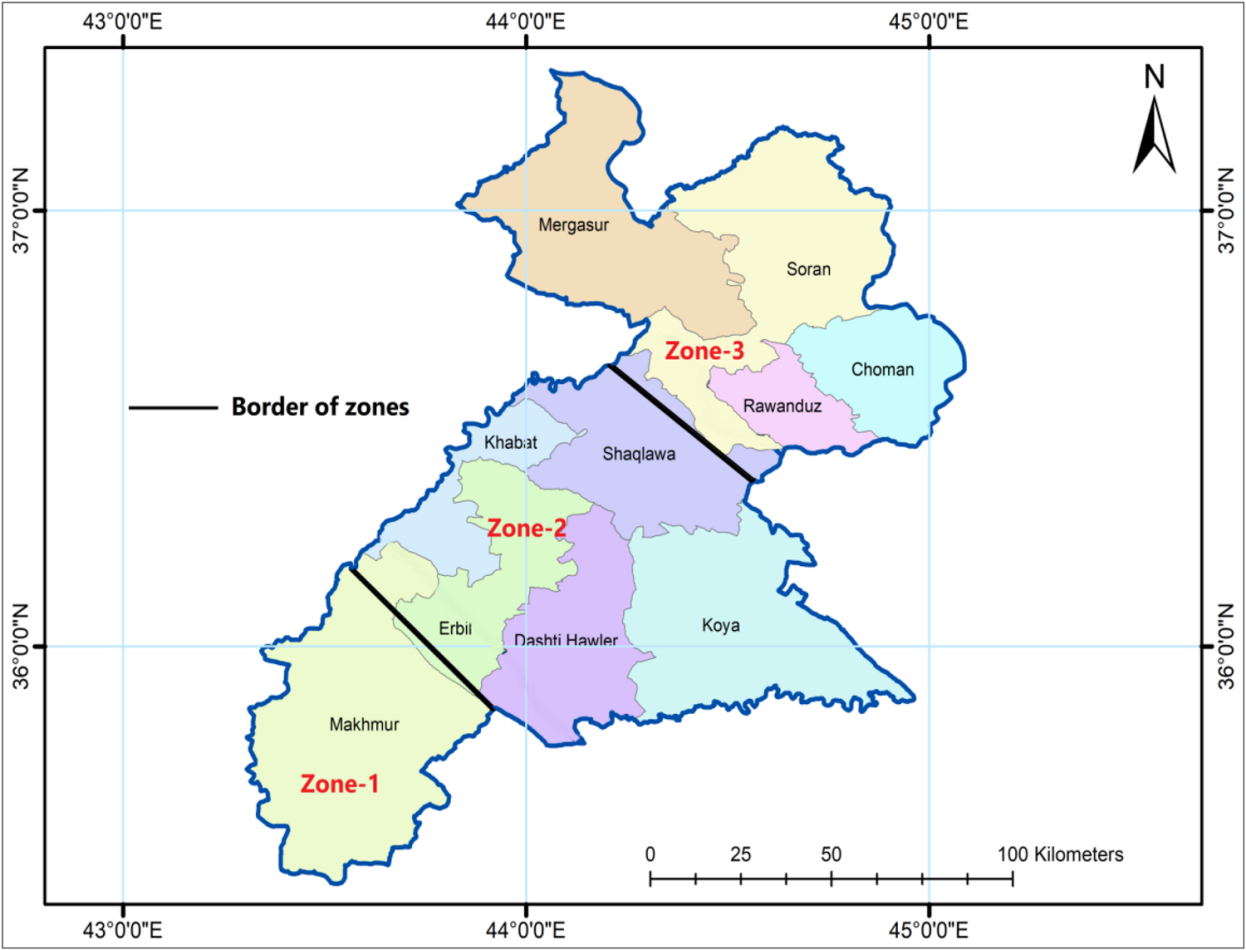
A map illustrating the division of Erbil Governorate into three distinct regions based on their physiographic features. Zone 1 (Southern Erbil): Qushtapa (#1), Makhmoor (#2), Dibaga (#3), Gwer (#4), Shamamk (#5). Zone 2 (Central Erbil): Erbil (#6), Khabat (#7), Bnaslawa (#8), Koya (#9), Taqtaq (#10), Bastora (#11). Zone 3 (Northern Erbil): Barzewa (#12), Harir (#13), Soran (#14), Shaqlawa (#15), Khalifan (#16), Choman (#17), Sidakan (#18), Rwanduz (#19), Mergasur (#20).

This study was conducted across the entire territory of Erbil Governorate, located in northern Iraq and one of its administrative regions. The topography of Erbil consists of two physiographic units: mountains and foothills. It is positioned between latitudes 36°12'11″ and 36°15'10″ N and longitudes 44°12'11″ and 44°15'10″ E, with elevations ranging from 400 to 500 meters above sea level (Figure 1). The area covers approximately 15,038.93 km^2^ and includes ten districts: Mergasur, Soran, Choman, Rawanduz, Shaqlawa, Khabat, and Dashti Hawler. The study was carried out throughout the entire Erbil Governorate in northern Iraq. The topography features two main physiographic zones: mountains and foothills. It lies between latitudes 36°12'11″ and 36°15'10″ N and longitudes 44°12'11″ and 44°15'10″ E. The elevation varies from 400 to 500 meters above sea level (see Figure 1). The region spans approximately 15,038.93 square kilometers and is divided into three zones: Zone 1 (Southern Erbil), Zone 2 (Central Erbil), and Zone 3 (Northern Erbil) [38]. Precipitation amounts are distributed heterogeneously across Erbil's spatial region; while mean annual precipitation reaches up to 430 mm, the majority falls in the northern parts. Mean daily temperature varies from 5°C during the winter season to 35°C during the summer months. However, this value rises to 50°C in southern portions [39]. According to Figure 2, the climate classification for this research area is Mediterranean (arid/semi‐arid) [40]. The natural conditions allow for division into three zones: Zone 1: arid, Zone 2: middle, and Zone 3: northern mountainous areas (Figure 3). Annual precipitation starts at 250 mm in southern regions but can exceed 1200 mm in high mountains bordering Iran's northeast side as well as Turkey's north [41]. The digital elevation model (DEM) depicted in Figure 1B reveals substantial variations in elevation throughout Erbil. The lower terrains, predominantly utilized for wheat cultivation (dark green hues), are situated in the southern and western sectors. At the same time, the higher altitudes exhibit decreased agricultural activity, indicated by the scarcity of cultivated land in the northeast. In Figure 1C, the data on annual precipitation (1998‐2021) illustrate a wide range from 240 to 410 mm/year in the southwestern areas to exceeding 1200 mm/year in the mountainous northern and northeastern regions. This distinct gradient underscores the influence of orographic factors, where elevated terrains receive augmented precipitation.

Figure 2 presents monthly climate data for Erbil (1998–2021), highlighting precipitation, relative humidity, potential evaporation (ETo), and temperature variations (maximum, minimum, mean). A clear seasonal pattern emerges, with most precipitation occurring from December to April and peaking in late winter and early spring. This period also features higher humidity and cooler temperatures. Conversely, summer (June–August) experiences minimal precipitation, high temperatures, sharply increased evaporation rates, and low relative humidity, emphasizing Erbil's arid to semi‐arid conditions. Figure 3 divides Erbil Governorate into three distinct physiographic zones. Zone 1 encompasses southern and southwestern lowlands and plains with modest elevations and drier conditions. Zone 2 includes a transitional region of foothills and mid‐elevation areas, experiencing higher precipitation than Zone 1 due to increased altitude. Zone 3 comprises mountainous northern and northeastern areas, characterized by significantly higher elevations and greater precipitation, demonstrating pronounced climatic and topographic diversity. Notably, agricultural land primarily occupies Zones 1 and 2, which experience lower and fluctuating precipitation. Therefore, understanding specific water requirements and conditions in normal, dry, and wet years is essential for effective agricultural management.

### 2.2 Meteorological Drought Indices

#### 2.2.1 Standardized Precipitation Index (SPI)

As this investigation relies on meteorological indices, it is of utmost importance to carefully select the appropriate index for comparing values across varying climate regions [42]. Consequently, the SPI index was utilized in a variety of analyses, including frequency and temporal‐spatial studies [43, 44, 45]. The SPI is computed by dividing the difference between the normalized seasonal precipitation and its long‐term seasonal mean by the standard deviation. The two‐parameter gamma distribution is used to fit probability distribution functions for annual precipitation in the region. It can be calculated using the formula:

(1) $$\mathrm{SPI}=\frac{Xij-Xim}{\sigma }$$

where **X_ij_** is the seasonal precipitation at the rain gauge station and the observation**, X_im_** is the long‐term seasonal mean, and σ is its standard deviation. Developed by McKee [46], the SPI has gained widespread attention over the past two decades due to its extensive theoretical development, robustness, and usefulness in drought assessments [47]. The SPI represents the number of standard deviations that a normally distributed random variable deviates from its long‐term mean, which in this case corresponds to the observed value [48]. The precipitation time series dataset is fitted to a gamma distribution, and then it is transformed into a normal distribution with the aid of an equal probability transformation [49]. The function of probability density defines the gamma distribution:

(2) $$g\left(x\right)=\frac{1}{\beta^{\alpha }\Gamma \left(\alpha \right)}x^{\alpha -1}e^{-\frac{x}{\beta }}$$

where ***α* and *β*** are shape and scale parameters, respectively; ***x*** is the amount of precipitation at a meteorological station; and **Γ(α)** is the gamma function. SPI calculation includes a gamma probability distribution fitted to a given frequency distribution of precipitation totals for a station. For each meteorological station, the ***α*** and ***β*** parameters of the gamma probability density function were estimated for twelve months and each year of the twenty years. To optimally estimate *α* and *β*, maximum likelihood solutions were used:

(3) $$\alpha =\frac{1}{4A}\left(1+\sqrt{1+\frac{4A}{3}}\right)$$

(4) $$\beta =\frac{\bar{x}}{\alpha }$$

(5) $$A=In\left(\bar{x}\right)-\frac{\sum \mathrm{In}\left(x\right)}{n}$$

where ***n*** is the number of observations.

The results from Equation (2) were used to calculate the cumulative probability of an observed precipitation event. Since the gamma function is undefined for ***x = 0*,** and a precipitation distribution may contain zeros, the cumulative probability, ***H(x)*,** is the extended gamma as shown in Equation (8):

(6) $$H\left(x\right)=q+1\left(1-q\right)G\left(x\right)$$

where ***q*** is the probability of a zero and ***G(x)*** is the cumulative probability of the incomplete gamma function. If ***m*** is the number of zeros in a precipitation time series, *q* can be estimated by ***m*/*n***. The cumulative probability is then transformed to the standard normal random variable, ***z*,** with a mean of zero and a variance of one, which is directly related to the distribution of the SPI [48]. When using DrinC software and considering the hydrological year (October‐September), the default calculation period commences in October with an annual initial calculation step. Categorized based on normalized data arising from SPI calculations, as illustrated through Table 1, are anomalous strength levels.

**TABLE 1:** SPI drought severity classes for wet and dry periods (McKee, 1995).

SPI	Class
2.0 or more	Extremely wet
1.5 to 1.99	Very wet
1.0 to 1.49	Moderately wet
0.99 to 0.99	Near normal
−1.0 to −1.49	Moderate drought
−1.5 to −1.99	Severe drought
−2.0 or less	Extreme drought

#### 2.2.2 Spatial Distribution of Precipitation Across the Study Area

The spatial distribution of yearly precipitation variability is determined using ArcGIS and the Kriging spatial interpolation method with data from 20 locations. In the KRI [50, 51]. The statistical foundation of Kriging methods generally makes them more suitable for hydrological applications than simpler interpolation techniques [52, 53]. The (CV) is a statistical measure used to quantify how much the precipitation deviates from the mean.

The CV is calculated using the following formula:

(7) $$CV=\frac{\;\mu \;}{\;\sigma }*100$$

where: σ = Standard deviation of precipitation, µ = Mean precipitation, Higher CV (%): Greater variability in precipitation (less predictable, more extreme fluctuations)., Lower CV (%): More stable precipitation distribution (consistent pattern) [54, 55, 56].

#### 2.2.3 Reference Evapotranspiration (ETo) and Crop Evapotranspiration (ETc)

The ETo is achieved through the utilization of the FAO Penman‐Monteith equation, which comprehensively incorporates physical and physiological factors that are integral to the evapotranspiration process [57]. Using this formula provides a reliable estimate of ETo based on the definition set by Penman‐Monteith. The formula details the variables needed to calculate potential evapotranspiration in millimeters per day.

(8) $$ETo=\frac{\left[0.408\Delta \left(\mathrm{Rn}\;-G\right)+\gamma \left(900/T\right)u_{2}\left(\mathrm{es}\;-\mathrm{ea}\right)\right]}{\left[\Delta +\gamma \left(1+0.34u_{2}\right)\right]}$$

where: ETo: Reference evapotranspiration (mm/day), Δ: Slope of the saturation vapor pressure curve (kPa/°C).

Rn: Net radiation flux density at the crop surface (MJ/m^2^/day), G: Soil heat flux density (MJ/m^2^/day), γ: Psychrometric constant (kPa/°C), T: Mean daily air temperature at 2 m height (°C), u_2_: Wind speed at 2 m height (m/s), es: Saturation vapor pressure (kPa), and ea: Actual vapor pressure (kPa) [57].

(9) $$ET_{c}=K_{c}\times ETo$$

ET_c_: Crop evapotranspiration (mm/day), ***K_c_***: Crop coefficient (dimensionless, varies by crop type and growth stage), and ETo: Reference evapotranspiration (mm/day)

(10) $$ET_{a}=ET_{c}\times K_{s}$$

ET_a_: Actual evapotranspiration (mm/day), ***ET_c_***: Crop evapotranspiration under standard conditions (mm/day), and ***K_s_***: Water stress coefficient.

In this research, CROPWAT 8.0 is a computational tool that facilitates the use of the FAO Penman‐Monteith equation and automates the complex calculations needed for practical irrigation planning and crop water requirement estimation. To assess the impact of drought on Erbil Governorate, the region was divided into three distinct zones based on its geographical characteristics, precipitation levels, temperatures, and agricultural activity (Figure 3). Zone 1 (Southern Erbil): Qushtapa (#1), Makhmoor (#2), Dibaga (#3), Gwer (#4), Shamamk (#5). Zone 2 (Central Erbil): Erbil (#6), Khabat (#7), Bnaslawa (#8), Koya (#9), Taqtaq (#10), Bastora (#11). Zone 3 (Northern Erbil): Barzewa (#12), Harir (#13), Soran (#14), Shaqlawa (#15), Khalifan (#16), Choman (#17), Sidakan (#18), Rwanduz (#19), Mergasur (#20).

#### 2.2.4 Autoregressive Integrated Moving Average (ARIMA) Model

Annual precipitation data from 20 meteorological stations across three zones of Erbil Governorate, Iraq, covering 1995–2022, were analyzed. Basic descriptive statistics (mean, standard deviation, coefficient of variation) were calculated to assess precipitation variability at both station and zone levels. Time series modeling was conducted using the Autoregressive Integrated Moving Average (ARIMA) method. The general ARIMA(p,d,q) equation is:

(11) $$\begin{array}{ccc} & & \mathrm{Yt}\;=\;c\;+\;\phi 1Y_{t-1}\;+\;\phi 2Y_{t-2}\;+\;L\;+\;\phi pY_{t-p}\;+\;\theta 1\varepsilon_{t-1}\\ & + & \theta 2\varepsilon_{t-2}\;+\;L\;+\;\theta q\varepsilon_{t-q}+\;\varepsilon t \end{array}$$

Yt represents the differenced time series (after applying order d), c is the constant term, ϕi are the autoregressive (AR) parameters (lagged values of the series), θj are the moving average (MA) parameters (lagged error terms), εt is white noise (error term), p is the number of autoregressive terms, d is the number of differencing operations applied to make the series stationary, and q is the number of moving average terms. Model selection was based on the Akaike Information Criterion (AIC), Root Mean Square Error (RMSE), and Mean Absolute Percentage Error (MAPE). The best fit for all zones was ARIMA(1,0,0), which simplifies to:

(12) $$Yt=c+\phi 1Y_{t-1}+\varepsilon t\;$$

Fitted ARIMA Modules

(13) $$\mathrm{Zone}1:\mathrm{Yt}=269.37+0.279Y_{t-1}+\varepsilon t$$

(14) $$\mathrm{Zone}2:\mathrm{Yt}=382.56+0.132Y_{t-1}+\varepsilon t\;$$

(15) Zone3:Yt=777.42+0.053Yt−1+εt

Forecasting was conducted for a 10‐year horizon, with 95% confidence intervals, to assess future precipitation variability and potential drought intensification across Erbil Governorate.

Time series trend analysis employed both parametric (simple linear regression) and non‐parametric methods (Mann‐Kendall, Spearman's rho, Kendall's τ_b_, and Pearson's correlation). Pettitt's test was used to detect abrupt changes and identify mutation points in potential evapotranspiration data. Spatial data processing was conducted using ArcGIS software [58, 59].

To perform a thorough analysis of drought dynamics, various temporal scales were used, including monthly, seasonal, and yearly resolutions. These scales were intentionally chosen to capture both short‐term fluctuations and long‐term trends, ensuring a comprehensive understanding of drought development in the Erbil Governorate. Additionally, a multi‐faceted approach combining indices such as the SPI, (CV), ETo, and other relevant metrics like ETa and ETc was adopted. This method enables a more detailed insight into drought dynamics by integrating short‐term and long‐term analyses, thereby improving the effectiveness of planning and resilience strategies [60]. By applying these methods, this research provides a broad and detailed view of drought characteristics and their impacts on the Erbil Governorate.

## 3 Results

### 3.1 The Precipitation Map Interpolation

**FIGURE 4:**
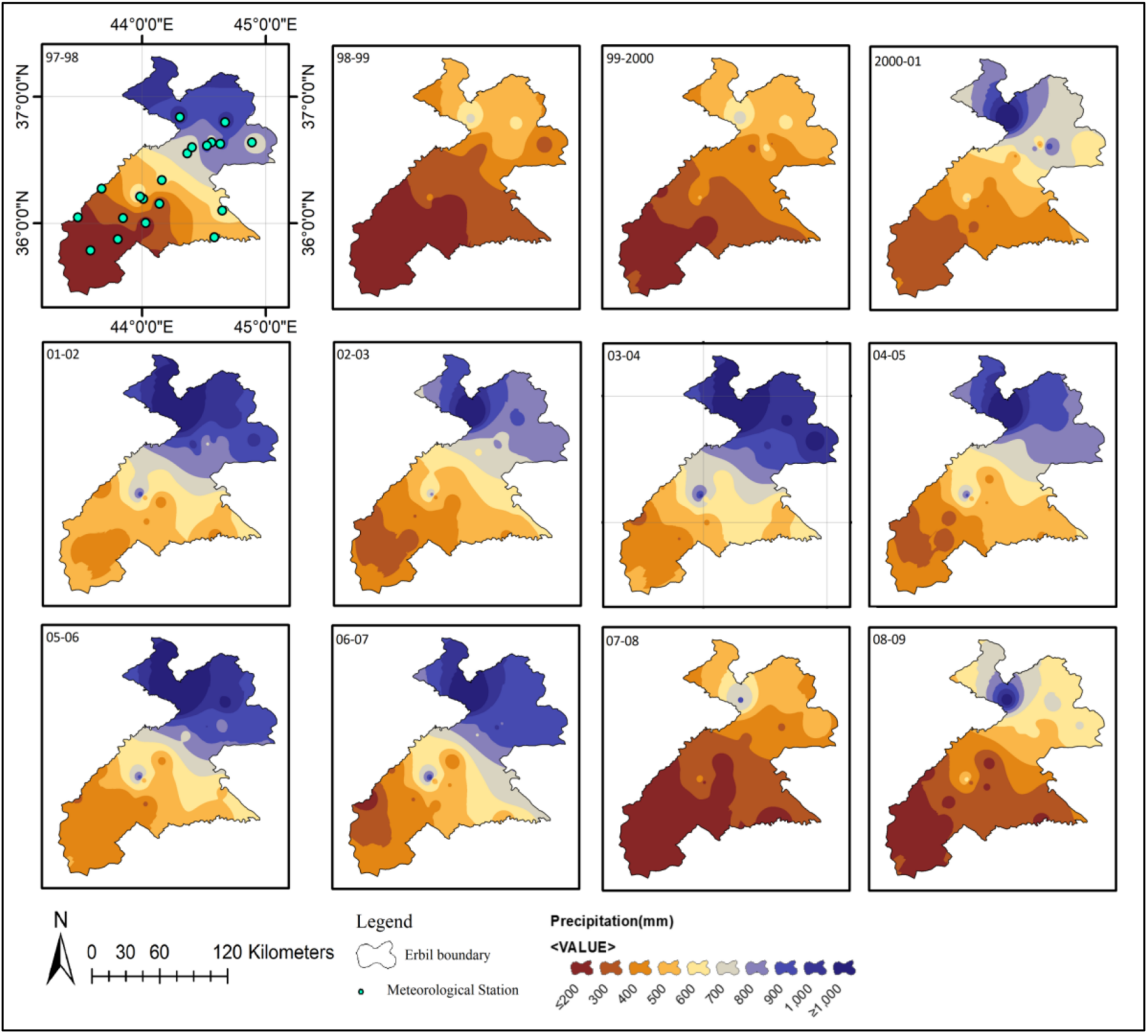
Spatiotemporal pattern of precipitation for 20 meteorological stations from 1998 to 2009.

**FIGURE 5:**
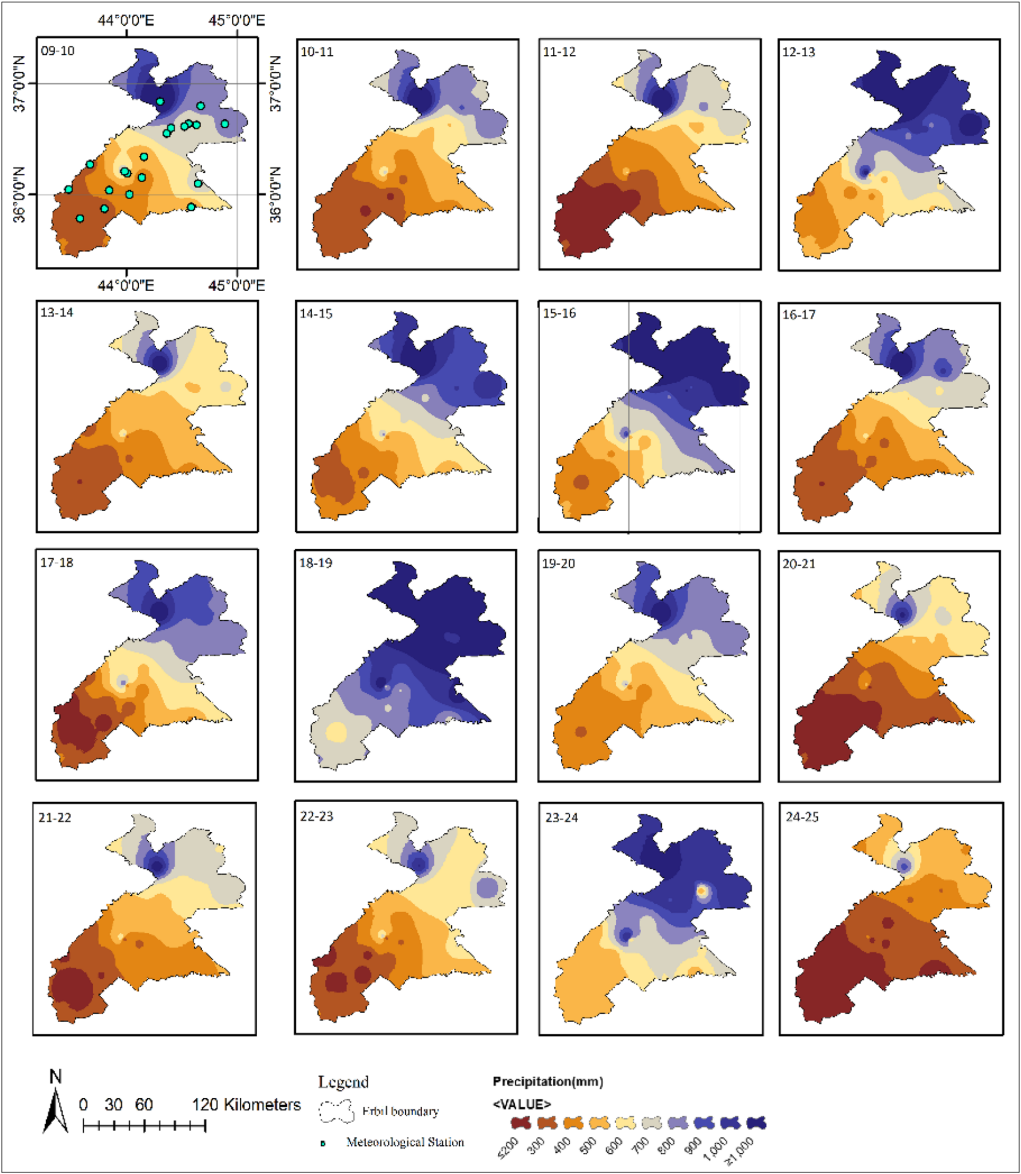
Spatiotemporal pattern of precipitation for 20 meteorological stations from 2010 to 2025.

In Erbil, 20 meteorological stations have been gathering data for 28 years. The data shows fluctuations in precipitation over time. Significant decreases in rainfall occurred during the periods between 1998–1999, 1999–2000, 2007–2008, 2008–2009, 2011–2012, 2020–2021, and 2024–2025 at most stations. However, there was a notable rise in precipitation during 2018–2019 and 2023–2024. The distribution of annual rainfall across Erbil over the past two decades, as recorded by these stations, was uneven, as shown in Figures 4 and 5. In 2021, arid regions like Zone 1 (Southern Erbil), Qushtapa (#1), Makhmoor (#2), Dibaga (#3), Gwer (#4), Shamamk (#5) experienced low annual average precipitation levels, less than 137.4 mm and 139.9 mm. Conversely, in 2019, the northern part of Zone 3 (Northern Erbil) Mergasur (#20) recorded the highest annual average rainfall, exceeding 2111.1 mm (Figures 4 and 5). The interpolation technique used in Figures 4 and 5 allows estimation of Precipitation in areas without direct measurements by using data from meteorological stations. The rainfall maps display the spatial and temporal patterns of precipitation across the Erbil region over several years. These maps, based on station data, provide a visual overview of variations in rainfall. The northern areas consistently receive more rain, while the southern regions are noticeably drier. Over time, a clear trend of decreasing rainfall becomes apparent.

In earlier periods (1997–1998 to 1999–2000), rainfall was more evenly distributed. From 2006, there has been a significant decrease in rainfall, especially in the southern and central regions. The maps from 2007–2008, 2008–2009, 2020–2021, and 2024–2025 show severe drought conditions. This declining trend suggests changes in climate patterns, potentially caused by global climate change, deforestation, or regional atmospheric shifts. The inclusion of meteorological stations in the first map (1997–1998) highlights the data points used for estimation. The map in Figure 4 illustrates recent spatial and temporal variations. In the Erbil Districts, average annual rainfall ranges from about 120 mm during dry periods to over 1300 mm in northern and mountainous areas. From 2010–2011 to 2012–2013, the southern regions experienced relatively low rainfall. This pattern persisted through 2013–2014, indicating a continued lack of significant rainfall in many parts of the area. However, from 2014–2015 to 2018–2019, rainfall increased considerably, affecting much of the region. During extreme droughts, rainfall in northern and mountainous areas can fall below 700 mm, resulting in more frequent and severe droughts. Understanding these rainfall patterns is essential for enhancing resilience and preparedness for future weather anomalies in the Erbil Districts.

The interpolation map depicted in Figure 5 has a similar format to Figure 4, but it covers more recent years and emphasizes spatial and temporal variations in precipitation. During the early years from 2010–2011 to 2012–2013, there was a noticeable trend of relatively low precipitation levels, particularly in the southern regions. This trend persisted through 2013–2014, indicating a consistent lack of significant precipitation in many parts of the area. However, a significant increase in precipitation was observed in the years 2014–2015, 2015–2016, 2018–2019, and 2023‐2024, with a large portion of the region experiencing higher precipitation. These fluctuations suggest a pattern of periodic wet and dry years rather than a continuous decline in precipitation.

The years 2020–2021 and 2024‐2025 show a return to drier conditions, similar to earlier drought years. This variability underscores the region's vulnerability to fluctuating precipitation levels, which can impact agriculture, water availability, and overall environmental sustainability.

### 3.2 Crop Area Maps

**FIGURE 6:**
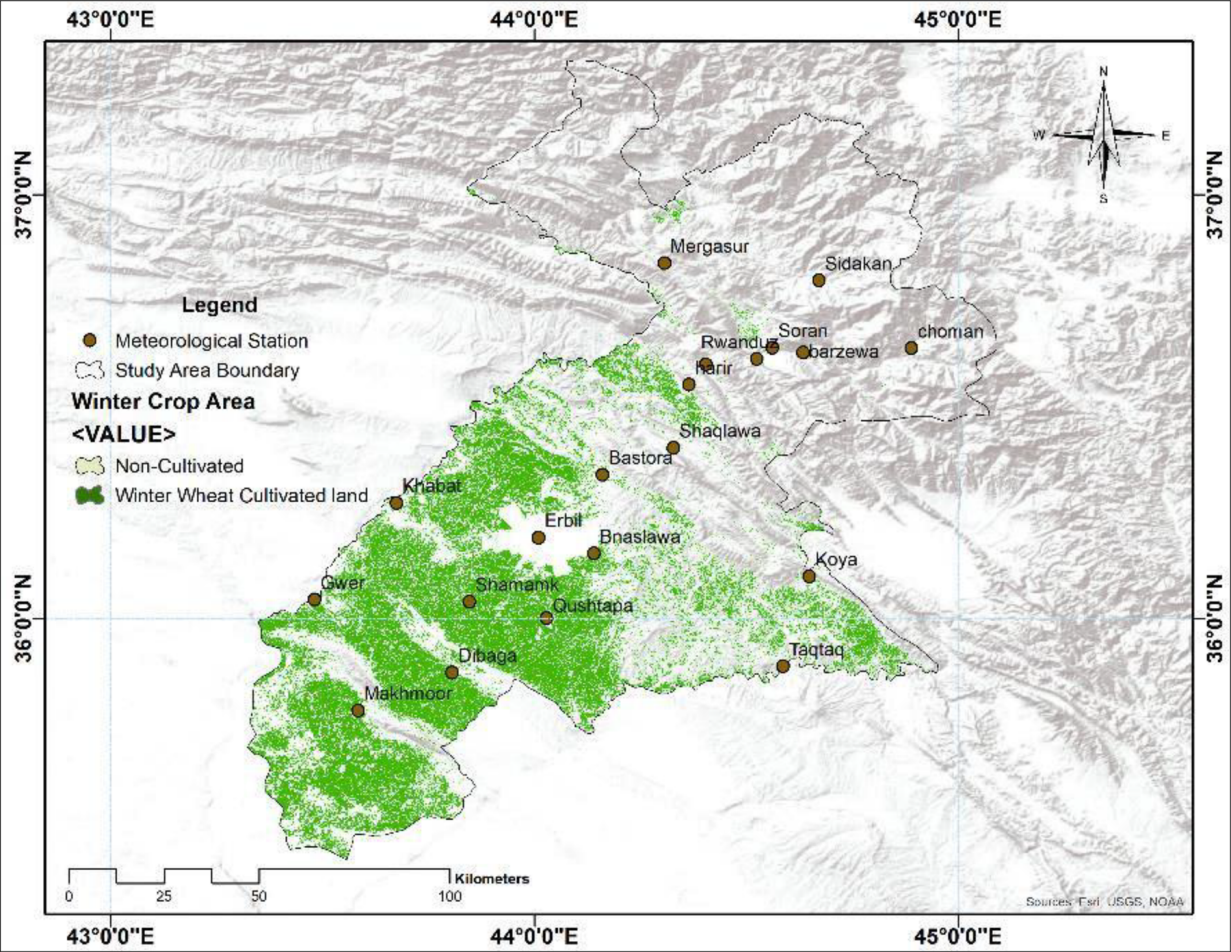
Spatial distribution of Winter wheat and barley cultivation in Erbil Governorate, 2024.

Table 2 and Figure 6 illustrate how agricultural and cultivated land are distributed across the study area, highlighting notable differences between zones. In the lowland plains, which include the districts of Zone 1 (Southern Erbil): Qushtapa (#1), Makhmoor (#2), Dibaga (#3), Gwer (#4), Shamamk (#5), and Zone 2 (Central Erbil): Erbil (#6), Khabat (#7), Bnaslawa (#8), Koya (#9), Taqtaq (#10), Bastora (#11), agriculture is highly concentrated in Zone 3 (#13Harir). These districts together account for nearly 70% of the total agricultural land (approximately 5,016 km^2^) and almost 90% of the total cultivated wheat and barley in 2024 (around 3,284 km^2^). The cultivation intensity here ranges from 65% to 77%, due to fertile soil, flat terrain, and easier access to irrigation. This zone serves as the main “grain basket” of the governorate, mainly involved in mechanized cereal farming. The mid‐elevation or transition zone, represented by Koysenjeq and Shaqlawa, accounts for roughly 20.6% of the total agricultural land (about 1,482 km^2^). Of this, nearly 593 km^2^ was used for wheat and barley cultivation in 2024, representing about 16% of total cultivation. The proportion of cultivated land in this area varies from 32% to 46%. Compared to the plains, farming here is more dispersed, with cereals grown alongside orchards, vineyards, and mixed‐use farms. This zone balances cereal crops and perennial plants, playing a vital supporting role.

**TABLE 2:** Distribution of Agricultural Land and Winter Wheat Cultivation by District in Erbil Governorate, 2024.

District name	Agriculture land (Km^2^)	Cultivated land wheat and barley‐2024 (Km^2^)	Cultivated land‐hectares	%Cultivated in agricultural land	District total area (Km^2^)
Choman	309.8	1.0	100	0.3	891.0
Qushtapa	817.7	630.0	63 000	77.0	1308.0
Erbil District Center	850.0	620.0	62 000	72.9	1132.0
Koya	867.2	396.0	39 600	45.7	2052.0
Maxmoor	2086.0	1466.0	146 600	70.3	2690.0
Mergaswr	356.8	31.0	3100	8.7	1977.0
Rawanduz	134.0	1.0	100	0.7	529.0
Shaqlawa	614.7	197.0	19 700	32.1	1473.0
Soran	738.4	11.0	1100	1.5	2130.0
Xabat	435.6	284.0	28 400	65.2	696.0
**Total**	**7210.2**	**3637.0**	363 700	**50.4**	**14878.0**

Highland and mountain districts such as Soran, Choman, Rawanduz, and Mergasur have very limited agricultural land, covering only about 712 km^2^, which accounts for less than 10% of the total area. Wheat and barley cultivation is also sparse, with just 44 km^2^ (1.2% of total cultivation) in 2024, and cultivation intensities ranging from 0.3% to 8.7%. The rugged terrain, steep slopes, and thin soils make these districts unsuitable for large‐scale cereal farming. Instead, they are better suited for forestry, grazing, orchards, biodiversity conservation, and eco‐tourism. Precipitation levels directly influence where crops can be cultivated. Figure 6 presents high‐resolution maps showing the distribution of wheat and barley in the Kurdistan Region of Iraq, specifically in Erbil. The map highlights cultivated areas, while white areas represent lands that are either uncultivated or used for other crops. As shown, the most cultivated areas are in the southwestern part of the area, while the least cultivated are in the northeast. The most cultivated lands are located in low‐rainfall zones; according to the Köppen climate classification and supporting research, Erbil's Zone‐1 is classified as dry, while Zone‐2 is semi‐arid. These zones are more suitable for agriculture due to their relatively moderate climate, which supports farming actices. In contrast, the uncultivated lands in Erbil are mainly in the mountainous Zone‐3, designated as the wet zone (Figures 4 and 5). Although Zone‐3 receives significantly higher precipitation, its rugged terrain, limited accessibility, and unfavorable topography prevent large‐scale farming, leaving much of this area undeveloped for agriculture with high rainfall, rock outcrops, forests, and shrubs.

**FIGURE 7:**
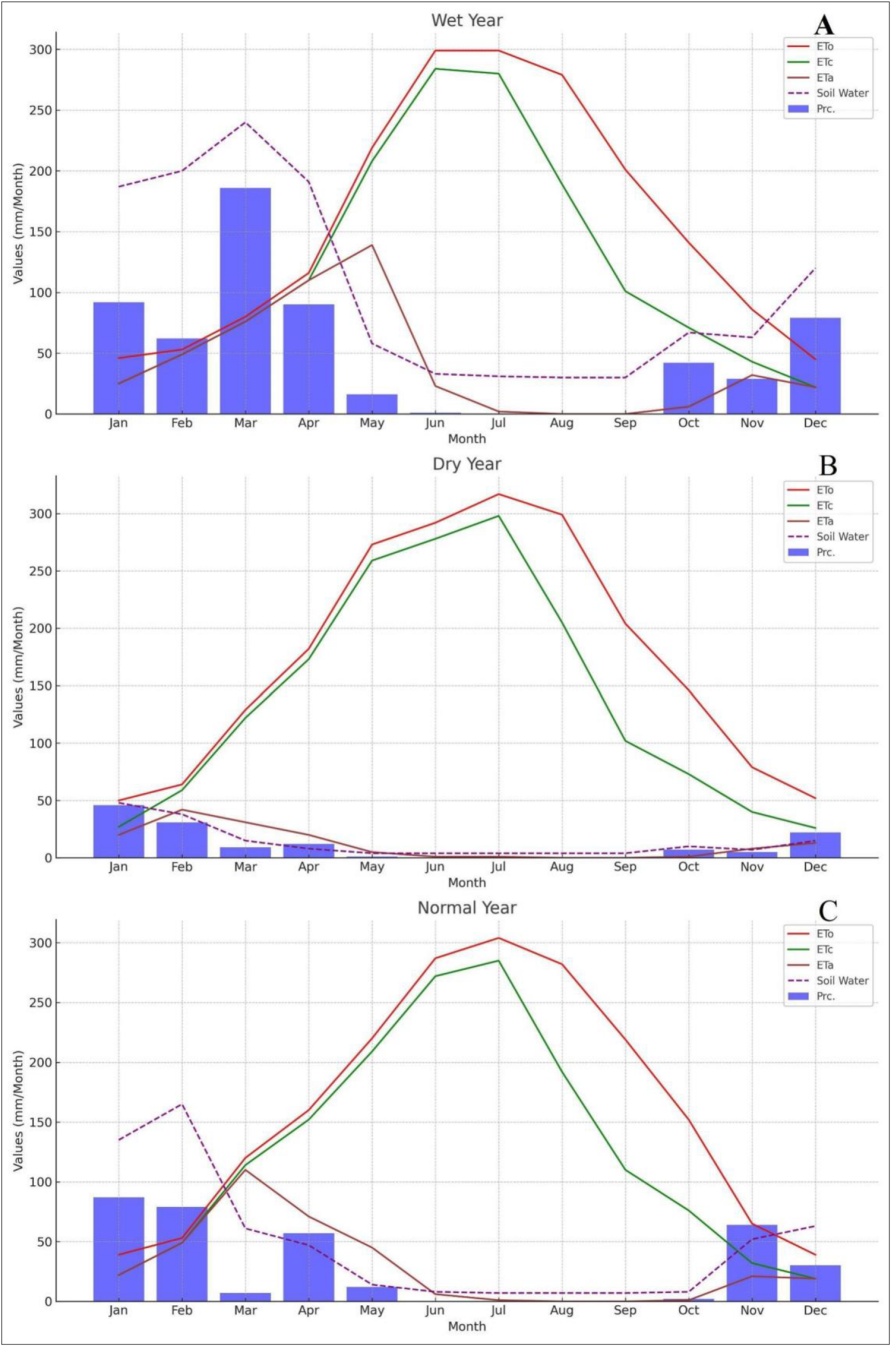
The graphs representing the monthly values of (Prc, ETc, and ETa) for each year type (wet, dry, standard) in Zone‐1.

The analysis of climate data for Erbil reveals distinct patterns in precipitation distribution across its zones. Zone‐1, characterized as dry, and Zone‐2, semi‐arid, experience lower but relatively stable precipitation compared to Zone‐3, which is classified as a wet zone and receives significantly higher rainfall. Despite the favorable precipitation conditions in Zone 3, much of its land remains uncultivated due to challenging terrain and limited accessibility. Conversely, Zones 1 and 2 are more suitable for agriculture, hosting most of the cultivated lands due to their moderate climate and relatively flat topography. Reference evapotranspiration (ETo), calculated using the FAO Penman‐Monteith equation, indicates higher values in the drier Zones 1 and 2, reflecting greater water demands for crops. In contrast, the wet Zone‐3 exhibits lower ETo values but remains underutilized for farming. The relationship between the Crop Area Map, Precipitation Map Interpolation, Evapotranspiration (ETo, ETc, and ETa) (Figure 7), and the Standardized Precipitation Index (SPI) provides an integrated view of agricultural and environmental dynamics in Erbil. Figure 6 shows that the cultivated areas in the southwestern part of Erbil (low rain‐fed lands, Zones 1 and 2) correlate with lower precipitation and a higher risk of droughts (Figures 4 and 5). This spatial distribution can be affected by variations in precipitation captured in the maps, which show spatiotemporal variability across Erbil, with significant decreases and increases in certain years impacting agricultural zones.

### 3.3 Reference Evapotranspiration (ETo), Crop Evapotranspiration (ETc), and Actual Evapotranspiration (ETa)

Precipitation levels and the spatial distribution of crop cultivation are key factors in determining variability in evapotranspiration rates. Figure 7 shows that even during normal or rainy years, there is a significant water deficit for winter wheat during critical crop growth months such as March and April. ‘Crop Deficit’ represents the difference between ETc and ETa, indicating water stress. For instance, in June of a dry year, the crop deficit is notably high (277 mm/m), signifying substantial water stress. In the wet year shown in Figure 7, precipitation was exceptionally high during the early months, peaking in March. Soil water storage was also elevated early in the year, providing enough moisture to support crop evapotranspiration. ET_0_ and ETc values reached their maximum around June and July, exceeding 250 mm/month, while ETa closely followed ETc until July, after which it decreased significantly, indicating declining soil moisture availability later in the year.

**FIGURE 8:**
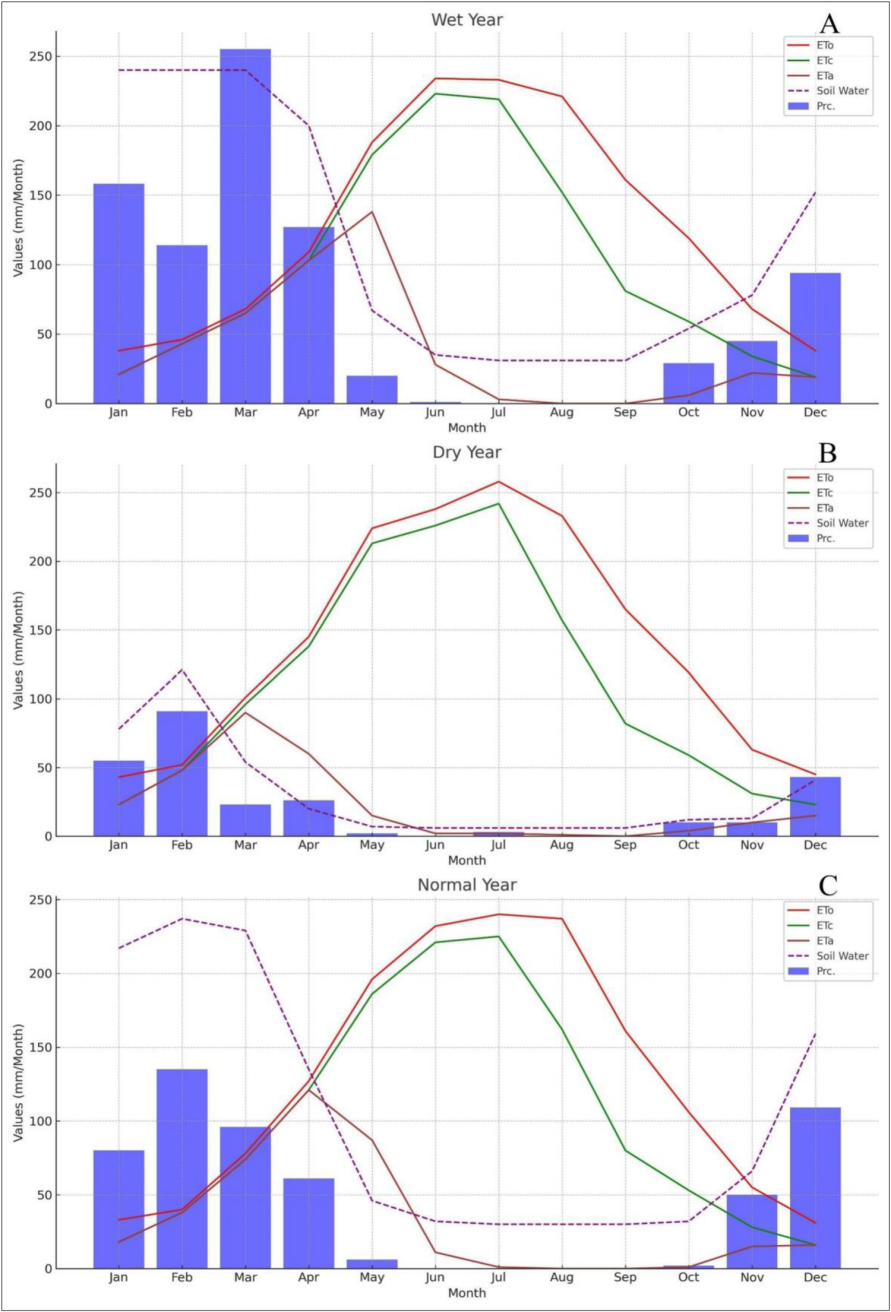
The graphs representing the monthly values of (Prc, ETc, and ETa) for each year type (wet, dry, standard) in Zone‐2.

The data shown in Figure 8 and Table A4 covers three distinct categories of years wet, dry, and normal within Zone 2, focusing on the soil water balance through various parameters including monthly precipitation (Prc.), ETc, and ETa. In wet years, high precipitation is evident, especially in the initial months such as January, February, and notably March (255 mm/m). This indicates an excess of precipitation contributing to elevated soil water levels. ETc and ETa in months like May and June, although ETc is considerably high (179 mm/m in May and 223 mm/m in June), ETa decreases significantly (138 mm/m in May and 28 mm/m in June), suggesting that despite the high potential evapotranspiration, the actual water use by crops is notably lower due to factors such as saturation, cloud cover, or cooler temperatures compared to standard conditions. Figure 8 and Table A4 show a clear difference between ETc and ETa during dry conditions, particularly from April to June. In this period, ETc peaks at 213 mm/m in May, while ETa is much lower at only 15 mm/m. Soil water levels stay consistently low throughout dry years, reflecting limited rainfall and high evapotranspiration requirements. These conditions highlight the critical importance of irrigation to support crops. Without adequate water supplementation, crops are likely to experience stress, reducing yield potential.

**FIGURE 9:**
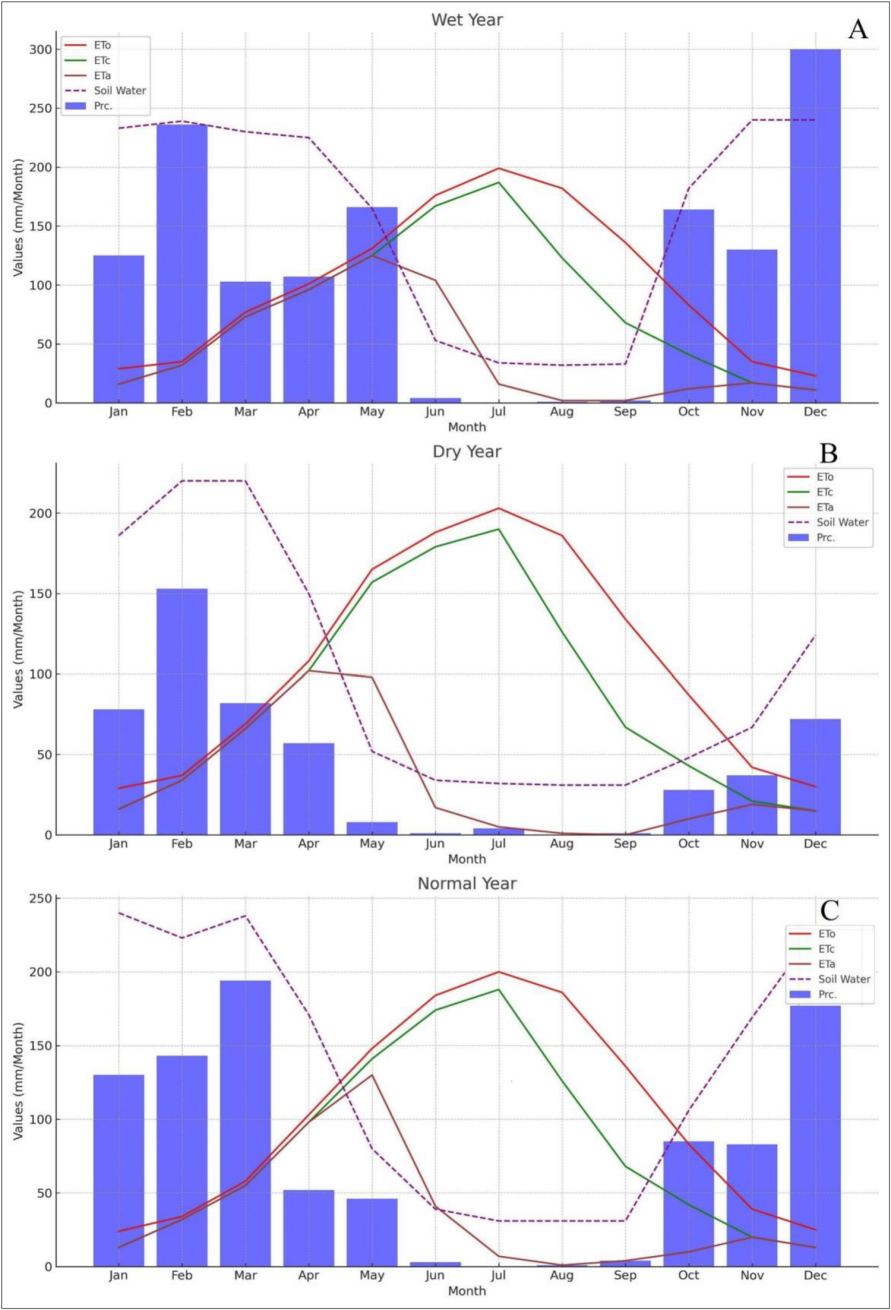
The graphs representing the monthly values of (Prc, ETc, and ETa) for each year type (wet, dry, standard) in Zone‐3.

Figure 9 and Table A5 display data for Zone 3 across wet, dry, and normal years, analyzing variability in precipitation (Prc.), evapotranspiration (ETc), and actual evapotranspiration (ETa). Recognizing this variability is crucial for effective water management and agricultural planning. In wet years, precipitation is significantly higher, especially in February (236 mm/m) and December (300 mm/m), indicating abundant moisture availability. This is important for managing water resources during heavy rainfall to avoid issues like waterlogging and nutrient leaching. However, a notable difference between ETc (167 mm/m) and ETa (104 mm/m) in June hints at possible water stress or suboptimal conditions affecting crop water uptake. Soil water levels are generally high, often reaching saturation or near‐saturation in many months, indicating good moisture supplies but also suggesting that drainage improvements might be needed during very wet periods. In dry years, precipitation levels are considerably lower than in wet years but remain above typical dry period levels, such as February (153 mm/m) and March (82 mm/m). The higher rainfall during these months could help ease some drought stress. ETc and ETa show a considerable gap in certain months, especially from May to July (ETc 157 mm/m vs. ETa 98 mm/m in May), indicating significant water deficits that may impact crop health and yields. In a normal year, precipitation follows a more consistent pattern, with peaks in March (194 mm/m) and December (204 mm/m), supporting steady soil moisture throughout the year. ETc and ETa are generally closely matched, with fewer significant differences than in dry years, implying that water supply usually meets crop demands under typical conditions. Nonetheless, June shows a large deficit (ETc 174 mm/m vs. ETa 41 mm/m), suggesting that even during normal years, some months may face water stress. Soil water levels are sufficient, reflecting effective water management without the extremes observed in wet or dry years. However, careful attention is needed during peak ETc months to prevent stress.

The spatial distribution of potential evapotranspiration across the study area shows a gradient from higher to lower values, with variations based on location and elevation. Monthly ETo patterns reflect these spatial trends, gradually decreasing toward the northern boundaries, with July displaying the most noticeable geographic differences in ETo compared to other months. Tables A3, A4 specifically support findings related to the southern region, where, despite increased evapotranspiration rates during periods of more intense drought, significant water deficits remain, especially in March and April. This region, heavily reliant on wheat/barley cultivation strategies, faces severe consequences due to insufficient precipitation relative to excessive transpiration/evaporation rates, significantly surpassing corresponding values of ETo (129), ETc (182), ETa (122), 173), (31), and 20, respectively. In general, Zone 1 exhibits moderate precipitation levels compared to the higher rates in Zone 3, aligning more closely with the levels observed in Zone 2. The distribution and volume of precipitation will significantly impact agricultural planning and water management strategies.

ETc and ETa. Similar to Zone 2, Zone 1 may experience high rates of evapotranspiration, especially during peak growing seasons. However, the actual crop evapotranspiration might be less impacted by large discrepancies during dry years, indicating better water retention or irrigation practices. Soil water is expected to show moderate variability, falling between the extremes seen in Zone 2 and Zone 3, which could suggest more consistent agricultural output across different year types.

Precipitation varies widely, ranging from high levels in wet years to minimal amounts in dry years, indicating significant variability that can significantly influence agricultural decisions each year. The differences between ETc and ETa, particularly in dry years, highlight potential water stress challenges that can impact crop yields. Precipitation often reaches or approaches capacity in wet years but is much lower in dry years. This indicates difficulties in managing water supplies amid changing climate conditions. As noted, Zone 3 experiences very high rainfall in wet years (236 mm/m in February and 300 mm/m in December), which can lead to waterlogging. The patterns are similar in wet and normal years but diverge in dry years, indicating a risk of water stress when precipitation is low. Soil water levels are high during wet and normal years, but managing excess water can be difficult and requires effective drainage. Climate variability in Zone 3 appears to be the most significant, with the most considerable fluctuations in rainfall and soil water, making water management especially challenging. In comparison, Zone 1 tends to have more moderate climate extremes, potentially offering more stability for farming. The significant differences in ETc and ETa across zones suggest that irrigation methods and crop selections may need to be highly customized. Zones with significant gaps, such as Zone 2 during dry years, might require advanced irrigation technology and strategies to manage water stress. Zone 3, characterized by high rainfall and a tendency for waterlogging, is quite different from the drier conditions usually seen in Zones 1 and 2. This requires different water management systems and infrastructure to boost agricultural productivity and resource efficiency.

### 3.4 Standardized Precipitation Index (SPI)

**FIGURE 10:**
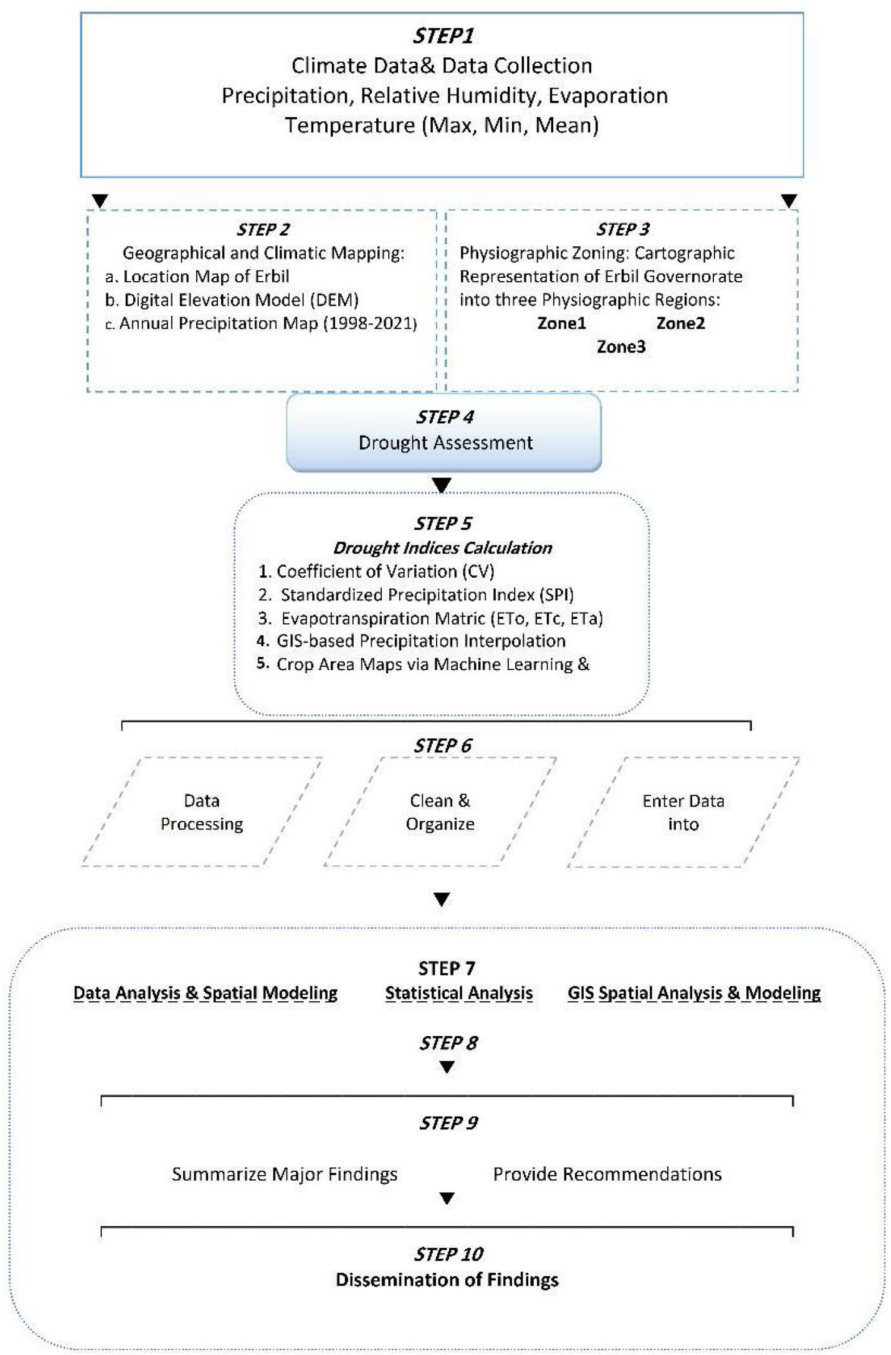
Flowchart of the methodology adopted in this study.

As illustrated in Figures 4 and 5 and Table A1, rainfall was primarily distributed across the northern, central, and southern parts of Erbil Governorate during various periods from 1998 to 2025. In the Erbil Districts, 20 meteorological stations were divided into three zones. Zone 1 (Southern Erbil, #1–#5), Zone 2 (Central Erbil, #6–#11), and Zone 3 (Northern Erbil, #12–#20) were assessed to evaluate spatiotemporal drought and wetness patterns from 1998 to 2025 using the Standardized Precipitation Index (SPI). From 1998 to 2000, the region experienced a basin‐wide dry spell, with most stations recording moderate to severe negative SPI. Conditions shifted during 2001–2005 toward a widespread wet phase that peaked around 2002–2004, when many stations observed sustained positive anomalies. A secondary basin‐wide drought occurred in 2007–2009. The majority of stations returned to negative SPI, with several reaching severe deficits, marking the driest multi‐year period since the late 1990s. Between 2011 and 2017, the pattern became more oscillatory, with short wet and dry periods and less spatial cohesion coherence. Figure 10 illustrates Erbil's climate‐based drought assessment. The workflow includes: collecting climate data (precipitation, humidity, evaporation, temperature); geographic and climatic mapping; zoning Erbil; assessing drought through indices (CV, SPI, evapotranspiration, precipitation, ML crop maps); GIS data processing and analysis; and synthesizing findings and recommendations to improve the accuracy of results.

This period lacked the persistent anomalies observed earlier, but localized extremes still occurred. The later record highlights a notably wet year in 2018–2019, when nearly all stations showed strongly positive SPI values, with several exceeding approximately +2 (e.g., #7, #3, #12). This was followed by renewed regional dryness in 2020–2021. Conditions then recovered in 2023–2024 before softening again into widespread mild negatives in 2024–2025. A clear north–south gradient is evident spatially. Zone 1 (Southern Erbil) is the most drought‐prone and volatile, with strong negatives during 1998–2000, 2007–2009, and again in 2020–2021 and 2024–2025. Zone 2 (Central Erbil) acts as a transition zone, closely following the overall domain signal but with slightly smaller fluctuations. Zone 3 (Northern Erbil) is comparatively resilient, showing stronger wet responses during rainy years (2002–2004, 2018–2019) and milder deficits during drought periods. At the station level, extremes align with these spatial patterns. For example, several stations exceeded SPI +2 in 2018–2019, underscoring that year's exceptional wetness, while stations in the south and center experienced severe negatives during 1998–2000 and 2007–2009. These peaks and dips reflect both regional influences and local sensitivities.

Drought‐resistant cropping, supplemental irrigation, and contingency plans should be prioritized in Zone 1, while Zone 3 can benefit from wet periods for recharge and strategic storage. A significant wet period occurred between 2001 and 2004, during which SPI values turned strongly positive. For example, Station #4 (Southern Erbil) peaked at +2.10 in 2002–2003, and Station #12 (Northern Erbil) reached +1.91 during the same period. Central stations also showed consistent wet signals, with Station #6 reporting +1.44 (2002–2003) and +1.28 (2003–2004). This indicates that 2002–2004 was one of the wettest multi‐year periods of the first decade.

The situation reversed between 2007 and 2009, with widespread drought impacting the region once again. Southern stations like #4 dropped to −1.21 (2006–2007), −1.11 (2007–2008), and −1.42 (2008–2009). Central stations such as #7 and #11 recorded very low SPI values of –1.98 and –2.14, respectively, during 2007–2008. Northern stations also showed effects, with #16 falling to –1.30 in 2007–2008. This confirms that 2007–2009 was another severe drought period. From 2011 to 2017, the region experienced alternating wet and dry years. For example, Station #2 (Southern Erbil) shifted from −1.28 (2011–2012) to +0.70 (2012–2013), then back to −0.30 (2013–2014). Station #15 (Northern Erbil) recorded +1.48 (2012–2013) but declined to −0.33 (2013–2014). These fluctuations indicate less spatial coherence and shorter‐lived anomalies compared to earlier periods. The 2018–2019 episode stands out as the wettest period in the entire record, with many stations exceeding SPI +2. For instance, Station #3 (Southern Erbil) reported +2.62, Station #7 (Central Erbil) reached +2.77, and Station #12 (Northern Erbil) recorded +2.62. This widespread wetness marked a basin‐wide pluvial year. After this peak, the region reverted to drought in 2020–2021, with southern stations like #1 recording −1.38, central stations such as #9 showing −1.21, and northern stations like #16 at −1.27, all indicating regional drought conditions, though less severe than the late 1990s or 2007–2009. In 2023–2024, many stations showed signs of a brief recovery toward wetter conditions; for example, Station #1 (Southern Erbil) recorded +1.89, and Station #20 (Northern Erbil) reached +2.08.

During 2024–2025, drought conditions re‐emerged across the Erbil Districts after a brief improvement in 2023–2024. In Zone 1 (Southern Erbil), all stations recorded negative SPI values, ranging from −0.89 at Stations #1 and #2 to −1.32 at Station #4, indicating mild to moderate drought, with Station #4 experiencing the highest dryness. Zone 2 (Central Erbil) experienced more severe droughts, with SPI values falling below −1.0 at nearly every station, reaching −1.49 at Station #6, −1.29 at Station #7, and as low as −2.05 at Station #10, signifying a severe drought level. Similarly, Stations #9 and #11 recorded −1.93 and −1.72, respectively, confirming widespread and intense drought across central Erbil. Zone 3 (Northern Erbil) also showed clear drought signals, with SPI values ranging from −0.57 at Station #12 to nearly −2.0 at Stations #15 (−1.90), #16 (−1.97), and #19 (−1.93). These figures indicate severe drought levels across much of the northern zone, with the most critical anomalies observed at highland stations (Tables 3 and 4).

**TABLE 3:** Spatiotemporal pattern of SPI drought and wet periods for 20 meteorological stations from 1998 to 2011 in Erbil Districts.

	Coordinate			Years													
	Station no.	longitude	latitude	1997–1998	1998–1999	1999 –2000	2000–2001	2001–2002	2002 –2003	2003–2004	2004–2005	2005–2006	2006–2007	2007–2008	2008–2009	2009–2010	2010–2011
**Zone 1** **(Southern Erbil)**	**#1**	44.028	36.001	1.44	−1.83	−1.59	−0.02	0.69	0.82	0.68	0.47	0.02	0.63	−0.79	−0.56	0.14	−0.99
	**#2**	43.583	35.783	−0.64	−1.76	−0.57	−0.02	1.10	1.52	1.06	0.34	0.74	0.33	−1.18	−1.00	−0.33	−0.20
	**#3**	43.805	35.873	−1.49	−1.85	−1.06	−0.31	0.85	1.25	0.43	0.12	0.93	0.48	−1.25	−0.60	−0.01	−0.69
	**#4**	43.481	36.045	−1.10	−1.60	−0.67	0.08	1.22	2.10	0.19	0.01	0.43	−1.21	−1.11	−1.42	−0.34	0.01
	**#5**	43.847	36.040	−0.70	−2.02	−0.60	−0.01	0.93	1.83	0.91	−0.27	0.19	0.14	−1.08	−0.89	0.08	−0.40
**Zone 2** **(Central Erbil)**	**#6**	44.009	36.191	−0.74	−2.12	−0.83	−0.16	0.67	1.44	1.28	0.78	0.75	0.49	−1.27	−0.50	0.28	−0.10
	**#7**	43.674	36.273	0.03	−1.32	−1.11	0.37	0.28	0.91	0.79	0.29	0.68	−0.06	−1.98	−1.19	−0.43	−0.08
	**#8**	44.140	36.154	−0.87	−2.05	−0.81	−0.19	0.10	1.10	1.19	0.50	0.56	0.48	−1.75	−1.23	−0.33	−0.39
	**#9**	44.648	36.099	0.26	−1.29	−0.81	−0.79	0.01	0.63	−0.30	−0.43	0.10	1.07	−1.81	−1.47	0.92	0.08
	**#10**	44.586	35.887	0.62	−1.04	−1.17	−0.49	0.03	0.38	0.53	0.19	0.29	0.56	−2.00	−1.81	0.57	−0.01
	**#11**	44.160	36.339	0.57	−0.95	−0.71	−0.16	0.78	0.68	0.63	−0.22	−0.23	−0.70	−2.14	−1.95	−0.03	−0.43
**Zone 3** **(Northern Erbil)**	**#12**	44.633	36.627	−0.34	−0.90	−0.27	0.20	1.05	1.91	1.04	−0.01	0.37	0.33	−0.57	−0.46	0.28	−0.11
	**#13**	44.365	36.551	0.28	−1.80	−1.29	−0.93	0.80	0.83	0.90	0.35	0.42	0.67	−1.80	−0.74	0.32	−0.53
	**#14**	44.561	36.638	−0.44	−0.96	−1.97	−1.28	0.90	1.06	0.93	0.38	0.95	0.74	−1.72	−0.68	−0.06	−0.72
	**#15**	43.985	36.209	0.38	−1.62	−1.27	−0.39	0.68	1.10	0.84	0.50	0.57	0.85	−1.75	−0.65	0.33	−0.20
	**#16**	44.404	36.599	−0.39	−2.12	−2.12	0.03	1.01	0.93	0.60	−0.05	0.73	0.59	−1.30	−0.61	0.23	−0.76
	**#17**	44.889	36.637	−0.27	−2.62	−1.65	−0.78	0.73	0.27	1.20	−0.01	0.32	0.63	−1.45	−0.55	0.03	0.26
	**#18**	44.671	36.797	0.71	−1.40	−1.36	−0.52	0.78	0.54	0.96	0.42	1.00	0.39	−1.86	−0.79	−0.10	0.04
	**#19**	44.525	36.612	1.09	−1.27	−0.49	0.30	−0.01	0.31	1.07	0.48	0.94	1.02	−2.02	−0.77	−0.22	−0.45
	**#20**	44.306	36.838	−0.95	−2.03	−1.95	0.00	0.74	0.17	0.57	0.28	0.98	0.23	−1.35	−0.59	0.62	0.22

**TABLE 4:** Spatiotemporal pattern of SPI drought and wet periods for 20 meteorological stations from 2011 to 2025 in Erbil Districts.

	Coordinate			Years													
	Station no.	Longitude	latitude	2011–2012	2012–2013	2013–2014	2014–2015	2015–2016	2016–2017	2017–2018	2018–2019	2019–2020	2020–2021	2021–2022	2022–2023	2023–2024	2024–2025
**Zone 1** **(Southern Erbil)**	**#1**	44.028	36.001	−1.46	0.62	0.02	0.37	0.90	−0.04	0.33	2.15	1.11	−1.38	0.37	−0.22	1.89	−0.89
	**#2**	43.583	35.783	−1.28	0.70	−0.30	−0.05	0.36	−0.30	0.13	2.25	1.35	−0.98	−1.83	0.07	1.34	−0.89
	**#3**	43.805	35.873	−1.08	1.19	−0.06	0.30	0.46	−0.02	0.21	2.62	1.28	−1.09	−0.54	−0.24	1.10	−0.99
	**#4**	43.481	36.045	−1.06	0.38	0.42	−0.01	0.62	0.14	0.17	2.19	1.26	−0.56	−0.38	−0.07	1.52	−1.32
	**#5**	43.847	36.040	−1.47	0.38	−0.33	0.19	0.76	0.04	0.38	2.56	0.85	−1.35	−0.21	−0.36	1.47	−1.08
**Zone 2** **(Central Erbil)**	**#6**	44.009	36.191	−1.15	0.71	−0.38	0.02	0.61	−0.31	0.27	1.97	0.65	−1.43	−0.55	−0.60	1.73	−1.49
	**#7**	43.674	36.273	−0.68	1.01	−0.17	0.42	0.78	−0.52	0.01	2.77	1.24	−1.01	−0.76	−0.44	1.43	−1.29
	**#8**	44.140	36.154	−0.71	0.97	−0.06	0.58	0.68	−0.47	0.46	2.07	0.69	−0.89	−0.38	−0.25	2.10	−1.12
	**#9**	44.648	36.099	0.03	0.63	−0.14	0.48	1.45	−0.32	0.60	2.42	1.00	−1.21	−0.58	0.35	1.07	−1.93
	**#10**	44.586	35.887	−0.40	0.81	0.15	0.52	1.44	−0.20	0.73	1.82	0.90	−1.56	−0.51	0.61	1.15	−2.05
	**#11**	44.160	36.339	−0.52	1.22	0.14	0.97	1.12	−0.19	0.71	2.00	0.76	−1.02	0.35	−0.45	1.51	−1.72
**Zone 3** **(Northern Erbil)**	**#12**	44.633	36.627	−0.75	0.54	−0.06	0.38	0.89	0.24	0.55	2.62	0.98	−0.70	0.03	−0.08	1.56	−0.57
	**#13**	44.365	36.551	−0.57	0.94	−0.83	0.57	1.04	−0.28	0.30	1.97	0.88	−0.83	−0.62	−0.10	1.75	−1.67
	**#14**	44.561	36.638	−0.63	0.62	−0.70	0.93	1.13	0.07	0.44	1.39	0.81	−0.44	−0.20	−0.60	1.81	−1.72
	**#15**	43.985	36.209	−0.86	1.48	−0.33	0.09	0.81	−0.57	0.00	1.79	0.43	−1.27	−0.48	−0.20	1.72	−1.97
	**#16**	44.404	36.599	−0.81	1.07	−0.33	0.39	1.10	−0.08	0.51	1.83	0.56	−0.69	−0.57	−0.28	1.84	−1.26
	**#17**	44.889	36.637	−0.40	1.28	−0.52	0.90	1.41	−0.53	0.48	1.47	0.47	−0.81	−0.29	0.26	1.54	−1.33
	**#18**	44.671	36.797	−0.32	0.67	−1.29	0.38	1.49	0.53	0.47	1.49	0.25	−0.73	−0.50	−1.27	1.81	−1.77
	**#19**	44.525	36.612	−0.96	1.05	−0.89	0.62	1.34	−0.22	0.23	1.36	0.47	−1.05	−0.80	−0.95	1.75	−1.93
	**#20**	44.306	36.838	0.05	1.53	−0.34	0.28	1.39	−0.05	−0.26	1.56	0.24	−0.74	−0.59	−0.80	2.08	−1.31

**FIGURE 11:**
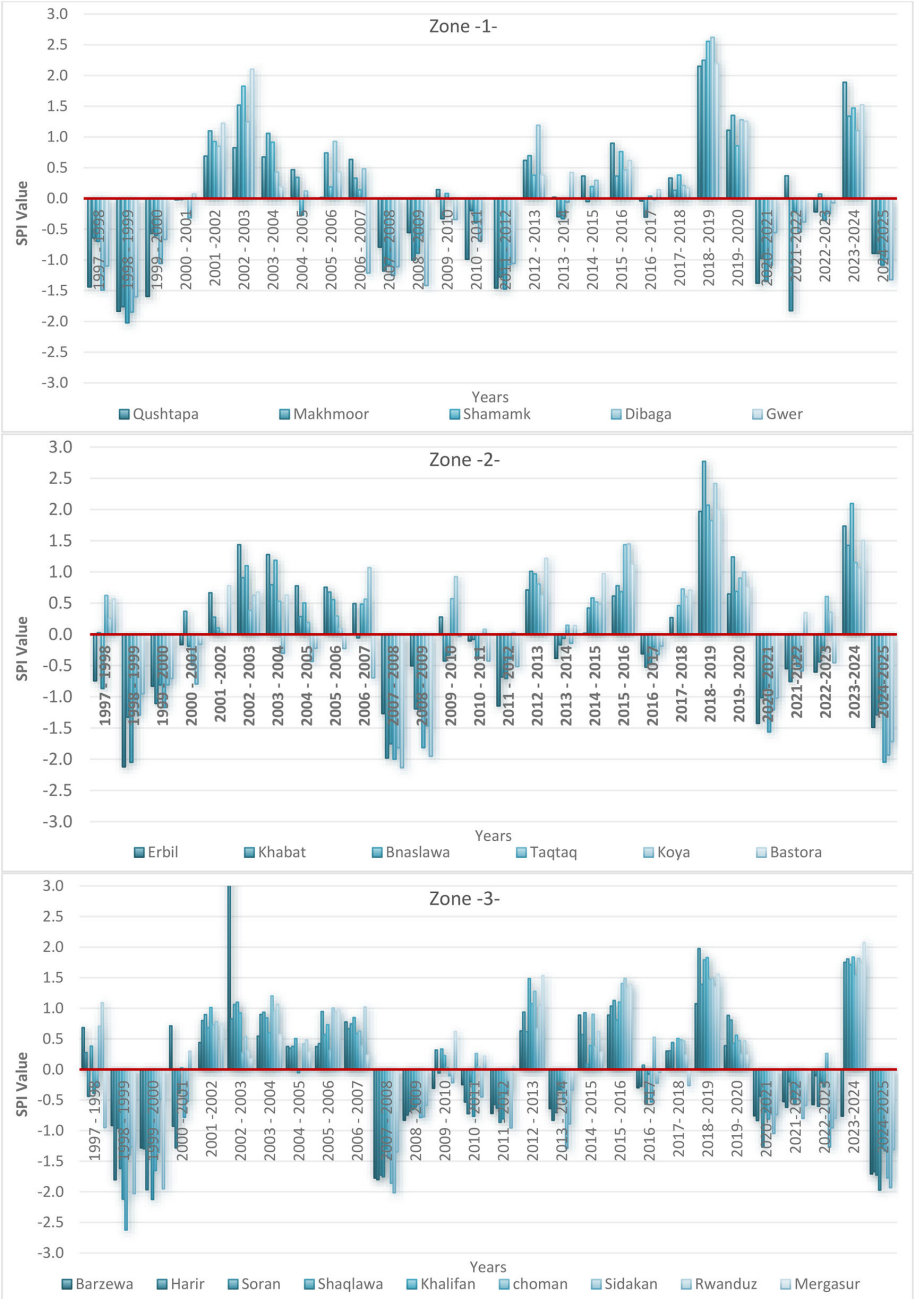
Spatiotemporal pattern of SPI drought and wet periods for 20 meteorological stations from 1998 to 2025. For 20 Meteorological Stations Across Three Zones: Zone 2 and Zone 3 in Erbil Governorate.

**FIGURE 12:**
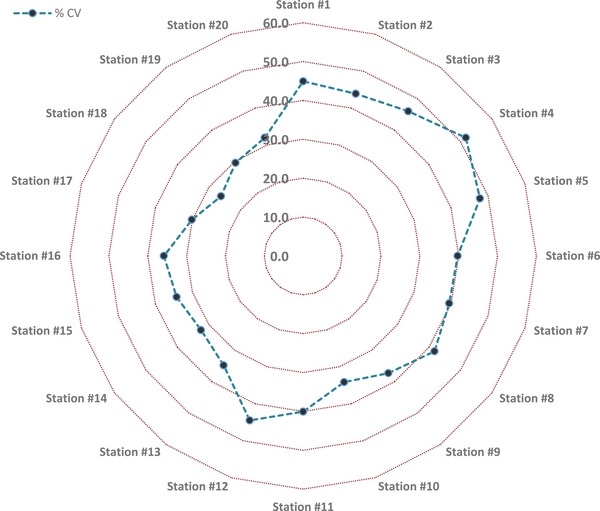
The %CV values of annual precipitation over 28 years for 20 meteorological stations across three zones in Erbil Governorate. (Zone 1 (Southern Erbil): Qushtapa (#1), Makhmoor (#2), Dibaga (#3), Gwer (#4), Shamamk (#5), Zone 2 (Central Erbil): Erbil (#6), Khabat (#7), Bnaslawa (#8), Koya (#9), Taqtaq (#10), Bastora (#11), Zone 3 (Northern Erbil): Barzewa (#12), Harir (#13), Soran (#14), Shaqlawa (#15), Khalifan (#16), Choman (#17), Sidakan (#18), Rwanduz (#19), Mergasur (#20)).

Table 5, Figure 11, and Figure 12 summarizes drought frequencies over 28 study years based on the SPI class. Extremely wet (≥ + 2.00), very wet (+1.50−1.99), moderately wet (+1.00−1.49), near‐normal (−0.99 to +0.99), moderate drought (−1.00 to −1.49), severe drought (−1.50 to −1.99), and extreme drought (≤ −2.00). Near‐normal conditions dominate across the network: most stations experience this class for 16–22 of the 28 years (approximately 57%–79%). Examples include #12 with 22 near‐normal years; #1 and #5 with 20; #9 with 18; and #2 and #4 with 16, indicating that more than half of the years at any station are neither strongly dry nor wet. Drought frequencies vary by intensity and location. Moderate drought (−1.00 to −1.49) occurs 1–7 times per station, with Station #4 (Southern Erbil) having the highest with seven years (25% of the record), while many others show 2–5 occurrences (e.g., #1, #2, #3, #6, #7, #18). Severe drought (−1.50 to −1.99) is less frequent but notable at several sites: #13, #14, and #15 each have three severe‐drought years, and #9, #10, #11, #18, #19, and #20 each have two. Extreme drought (≤ −2.00) is rare but occurs at ten stations; the highest counts are at #10 and #16, with two years each, and there are single occurrences at #5, #6, #8, #11, #17, #19, and #20.

**TABLE 5:** The frequency of the drought SPI index at 20 weather stations over 28 years, categorized by SPI class.

SPI Class								
—	—	Extremely wet	Very wet	Moderately wet	Near normal	Moderate drought	Severe drought	Extreme drought
Zone no.	Station no.	2.00 or more	1.5 to 1.99	1.00 to 0.49	0.99 to −0.99	−1.00 to −1.49	−1.50 to −1.99	2 or fewer
**Zone 1** **(Southern Erbil)**	**#1**	1	1	1	20	4	1	—
	**#2**	1	1	4	16	4	2	—
	**#3**	1	—	4	17	5	1	—
	**#4**	2	1	1	16	7	1	—
	**#5**	1	1	1	20	4	—	1
**Zone 2** **(Central Erbil)**	**#6**		2	2	19	4	—	1
	**#7**	1	—	2	19	5	1	—
	**#8**	2	—	2	20	2	1	1
	**#9**	1	—	4	18	3	2	—
	**#10**	—	1	2	19	2	2	2
	**#11**	1	1	2	20	1	2	1
**Zone 3** **(Northern Erbil)**	**#12**	1	2	2	22	2	—	—
	**#13**	—	2	1	21	1	3	—
	**#14**	—	1	3	20	1	3	—
	**#15**	—	2	2	19	2	3	—
	**#16**	—	2	3	19	2	—	2
	**#17**	—	1	3	20	2	1	1
	**#18**	—	1	3	18	4	2	—
	**#19**	—	1	6	16	2	2	1
	**#20**	1	2	1	20	2	1	1

Wet extremes appear infrequently and concentrate at specific sites. Extremely wet years (SPI ≥ +2.00) occur 0–2 times per station; #4 and #8 each have 2, and many stations have 1 (e.g., #1, #2, #3, #5, #7, #9, #11, #12, #20). “Very wet” years (+1.50−1.99) are slightly more widespread, especially in the northern zone: #12, #13, #15, #16, #20 all record two such years, whereas southern and central stations typically record 0–1. Viewed by zone, the pattern is clear. Zone 1 (Southern Erbil) shows the fewest high‐intensity droughts (only occasional severe and extreme events), but it has the highest recurrence of moderate drought, led by #4 (7) and #1/#2/#5 (4 each). Zone 2 (Central Erbil) exhibits greater volatility, including the network's most drought‐prone station, #10, which combines two severe and two extreme drought years (4/28 ≈ 14%). Zone 3 (Northern Erbil) experiences the widest swings: it has several stations with multiple severe droughts (#13, #14, #15: 3 each) and also concentrates many very/extremely wet counts (e.g., #12, #15, #16, #20), indicating stronger bimodality in the north. In practical terms, Table 5 confirms a backbone of near‐normal years at all sites, punctuated by episodic drought and pluvial bursts. Planning should therefore be zone‐sensitive: prioritize drought‐proofing and supplemental irrigation in Southern Erbil (frequent moderate deficits), invest in risk buffers and flexible allocations in Central and Northern Erbil, where severe to extreme drought (up to 2 years at #10 and #16) and very/extreme wet years (up to 2 at several stations) make inter‐annual variability a stronger operational challenge.

### 3.5 Coefficient Variation of Annual and Monthly Precipitation

**FIGURE 13:**
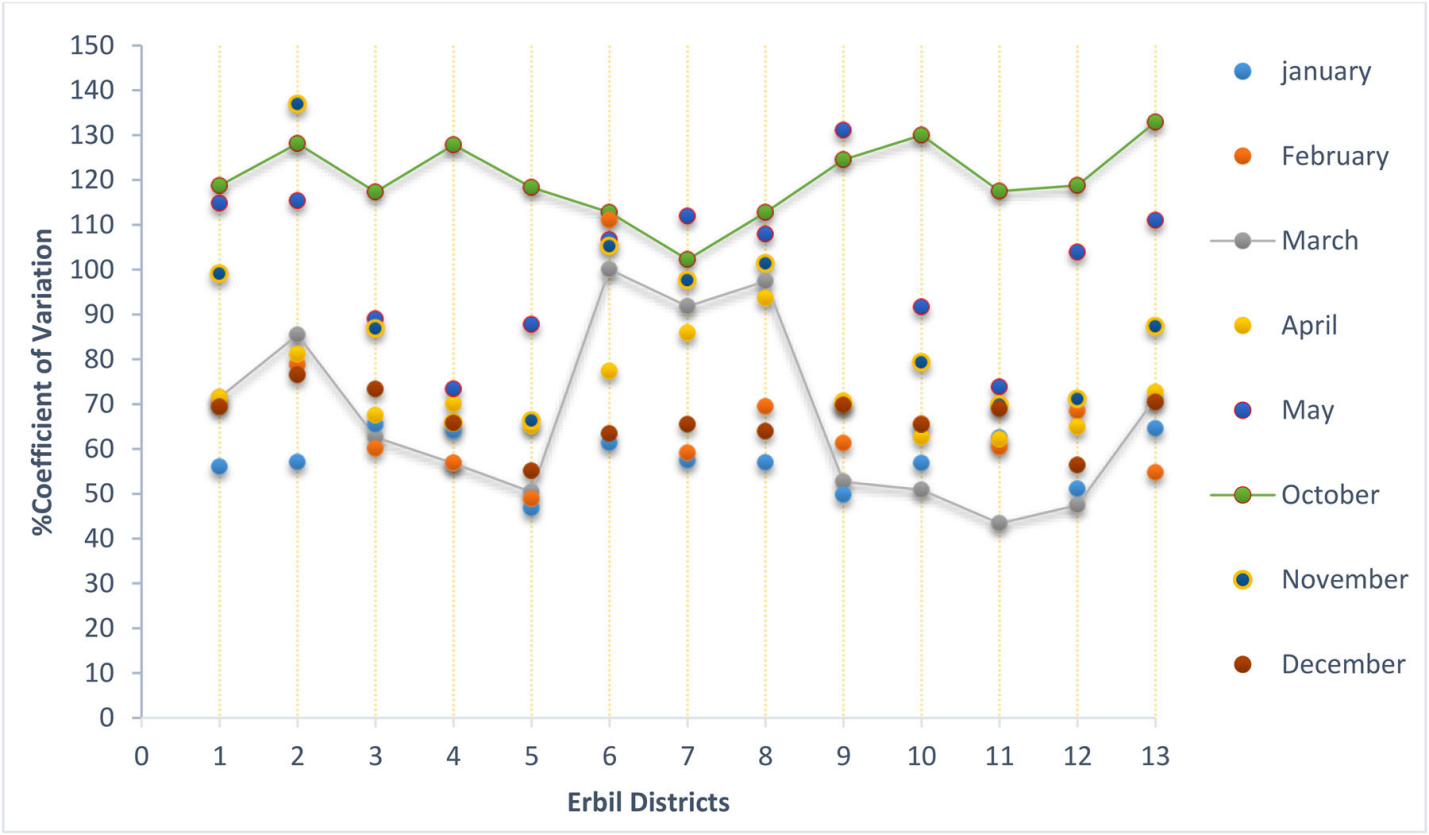
The mean and coefficient of variation (CV%) for monthly precipitation over 24 years for thirteen selected meteorological stations across the Erbil Governorate, namely: Erbil = 1, Qushtapa = 2, Shaqlawa = 3, Mergasur = 4, Sidakan = 5, Maxmur = 6, Gwer = 7, Xabat = 8, Xalifan = 9, Soran = 10, Choman = 11, Rwandz = 12 and Bastora = 13.

Figure 13 shows the coefficient of variation (%CV) of annual precipitation across 20 meteorological stations in Erbil Governorate. The %CV of yearly precipitation ranges from 26.1% at Station #18 to 51.7% at Station #4. This variation highlights the difference between relatively stable mountainous areas and highly variable lowland sites. The network average is around 38%, placing the governorate in the moderate to high variability range typical of semi‐arid climates. Such variability indicates the strong impact of year‐to‐year climate fluctuations on rainfall patterns.

Only two stations, #18 (26.1%) and #19 (29.6%), fall into the low variability category. These are situated in the mountainous northeastern part of the governorate and receive the highest average rainfall totals (706–823 mm annually). Their stability, combined with high rainfall amounts, makes them the least drought‐prone locations and the most reliable for rainfed farming. Stations such as #9, #10, #13, #14, #15, #16, and #20 (CV = 32%–37%) represent the moderate variability category. These sites have significant rainfall averages ranging from 494 mm to 1365 mm, supporting good agricultural potential. However, the year‐to‐year fluctuations still require careful water resource planning, as occasional shortfalls may affect productivity. Station #20 is especially notable, with the highest rainfall average (1365 mm) and a moderate CV of 32%, confirming the stabilizing influence of orographic rainfall.

The majority of stations fall into the high variability group (40%–50%), including #1, #2, #3, #5, #6, #7, #8, #11, and #12. These sites are distributed across the central plains and foothills. They generally receive moderate rainfall (240–710 mm annually) but experience much greater fluctuations from year to year. This group is especially vulnerable to alternating drought and flood cycles, which complicates both agriculture and water storage management. Station #4 (CV = 51.7%) stands out as the most variable site in the network. With a relatively low annual mean of 256 mm, this station is highly prone to drought and represents the most fragile environment for rainfed farming. The combination of low rainfall and extreme variability raises the risk of water stress and crop failure. Overall, mountainous stations in the northeast (e.g., #18, #19, #20) record the highest rainfall with relatively low variability, reflecting the stabilizing influence of elevation. Conversely, the central and southern plains (e.g., #1–#8) show high variability, lower rainfall, and a greater risk of drought. The foothill and southern transitional zones (e.g., #13 and #16) have moderate variability and rainfall. This spatial pattern indicates that future water resource planning should focus on irrigation and drought mitigation in the plains while utilizing the stability of mountain water resources for long‐term agricultural security.

**FIGURE 14:**
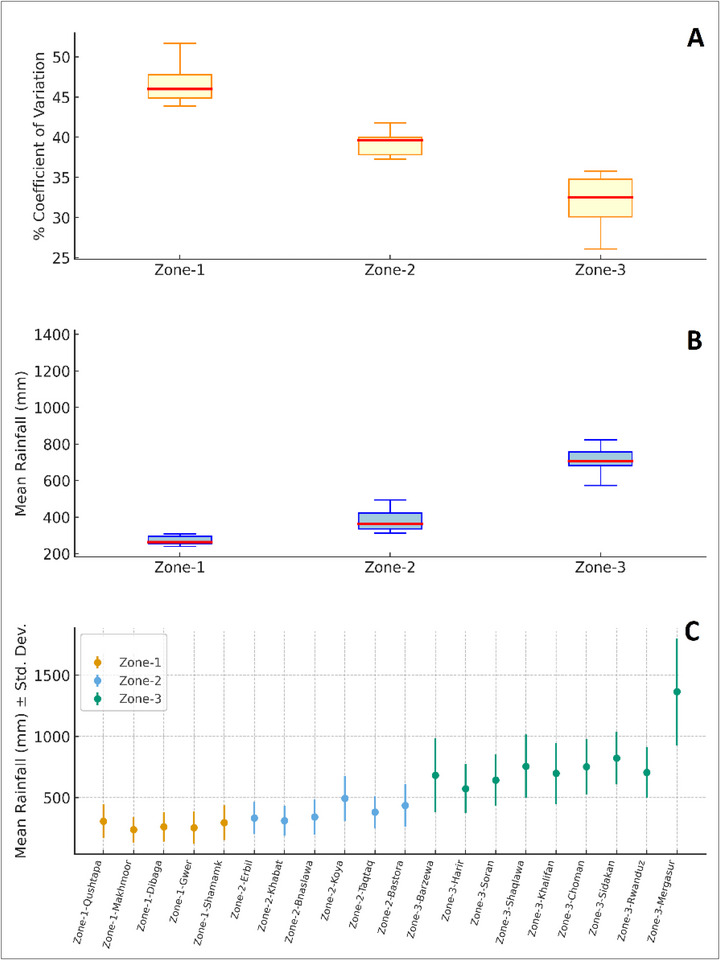
Precipitation patterns across three zones. (A) Boxplot of precipitation variability expressed as the(%CV), in Zone‐1, Zone‐2, and Zone‐3. (B) Boxplot of mean annual precipitation by zone, highlighting the gradient from dry lowland areas (Zone‐1) to wetter highlands (Zone‐3). (C) Mean precipitation with standard deviation for each location, from Zone‐1 to Zone‐3.

Figure 13 shows the spatial distribution of (CV) values for each month from January to December. Among all the stations analyzed, Erbil has the highest CV value, indicating a wider spread of data points around the mean and greater variability in precipitation during this period. Nearby areas such as Qushtapa, Maxmur, and Gwer also display high CV values, ranging from 115% to 128%. This consistent pattern across the region suggests less uniformity and more unpredictability in precipitation patterns. The study also highlights a clear downward trend in both monthly and seasonal precipitation within the Erbil region over time. These changes have important implications for water resource management and agriculture in the area. Notably, the southern parts of Erbil show significant year‐to‐year fluctuations in precipitation's CV%, with areas that usually experience low levels showing exceptionally high CV values (as shown inTable A2 in the Appendix). These results indicate that regions with minimal rainfall are more vulnerable to extreme variability from year to year. Figure 14 depicts Erbil as a central hub with the highest (CV), emphasizing increased fluctuations in precipitation from May through October. Similarly, the larger southwestern region experiences considerable variation, underscoring the importance of careful evaluation and flexible strategies when managing water resources and addressing the impacts of changing precipitation trends.

Figure 14 presents the analysis of precipitation across the three zones, revealing a clear spatial gradient in both amount and variability. Zone 1 recorded the lowest average annual precipitation (≈240–310 mm), indicating consistently dry conditions. Zone 2 represented an intermediate regime (≈330–490 mm), while Zone 3 had the highest averages, ranging from 570 mm to over 1300 mm in Mergasur, which stood out as a noticeable outlier. This indicates that precipitation gradually increases toward the highland areas with significant spatial differences. The variability analysis, displayed as standard deviation and summarized with box plots, highlighted distinct differences among the zones. In Zone 1, total precipitation was low, but variability remained consistently high, indicating unreliable rainfall despite its narrow range. Zone 2 had wider precipitation distributions, with moderate year‐to‐year variations, suggesting more balanced yet still variable conditions.

Zone 3 showed the greatest variation in precipitation. While most sites consistently reported higher totals, some experienced notable fluctuations, indicating localized instability in the highlands. The coefficient of variation (%CV) provided deeper insight into precipitation stability. Zone 1 displayed the highest variability, with median values around 46%, suggesting that precipitation in this zone is both scarce and unreliable. Zone 2 had moderate stability, with median %CV values near 39%, reflecting more dependable conditions suitable for rainfed agriculture. Zone 3 exhibited the lowest median variability (≈32%). Still, the wide range (26%–45%) indicated that some sub‐locations, like Sidakan and Choman, are highly stable, while others, such as Barzewa, are prone to fluctuations. These results have direct implications for water management and agricultural planning. Zone 1 is the most vulnerable, requiring urgent interventions such as irrigation, water harvesting, and the promotion of drought‐resistant crops. Zone 2, with moderate stability, offers opportunities for implementing climate‐smart agriculture and improving water use efficiency. Zone 3, though generally wetter and more reliable, still needs site‐specific adaptation strategies, as extreme variability in certain areas can hinder agricultural productivity and resource security.

### 3.6 Autoregressive Integrated Moving Average (ARIMA) Model

**FIGURE 15:**
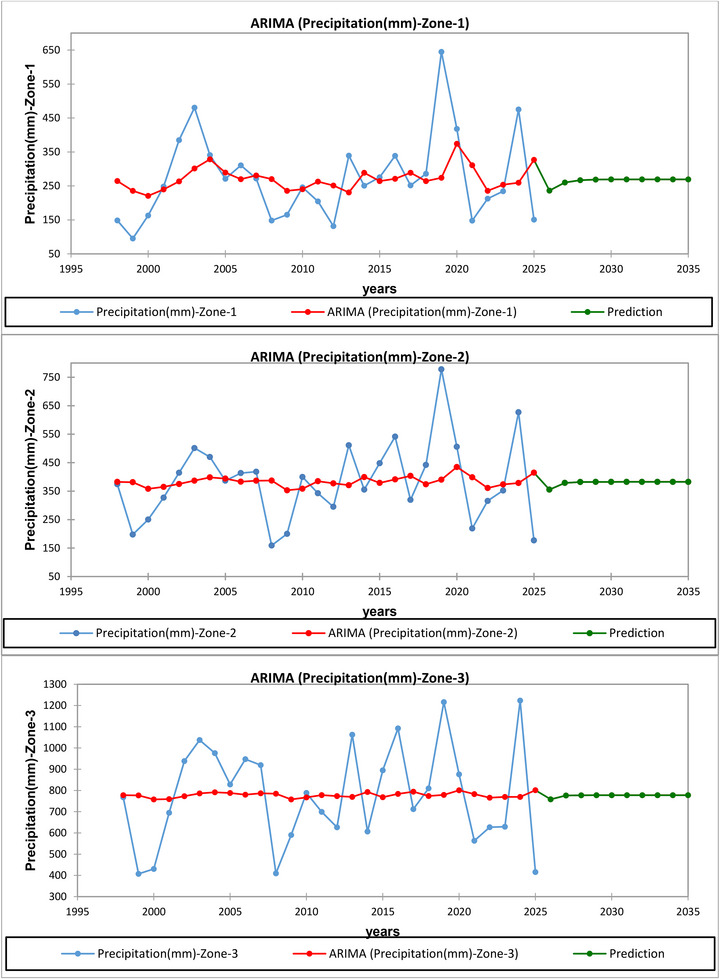
Historical Trends and ARIMA Forecasts of Annual Precipitation (mm) for Meteorological Stations Across Three Zones in Erbil Governorate (1998–2035): Zone 1 (Southern Erbil): Qushtapa (#1), Makhmoor (#2), Dibaga (#3), Gwer (#4), Shamamk (#5) Zone 2 (Central Erbil): Erbil (#6), Khabat (#7), Bnaslawa (#8), Koya (#9), and Taqtaq (#10). Zone 3 (Northern Erbil): Barzewa (#12), Harir (#13), Soran (#14), Shaqlawa (#15), Khalifan (#16), Choman (#17), Sidakan (#18), Rwanduz (#19), Mergasur (#20).

**FIGURE A1:**
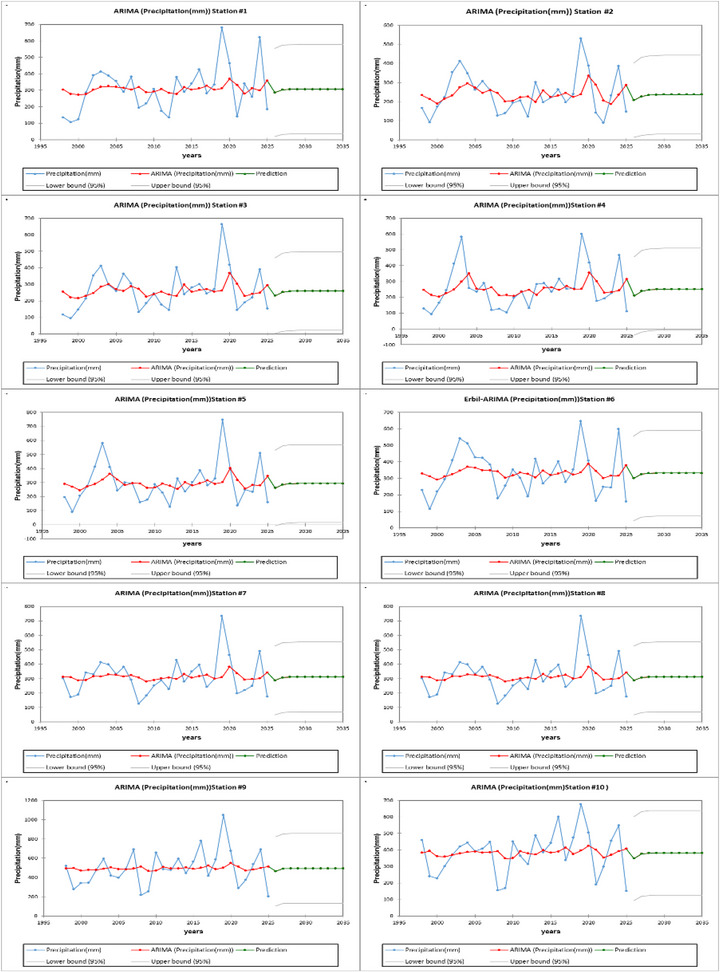
Historical Trends and ARIMA Forecasts of Annual Precipitation (mm) for Meteorological Stations Across Three Zones in Erbil Governorate (1998–2035): Zone 1 (Southern Erbil): Qushtapa (#1), Makhmoor (#2), Dibaga (#3), Gwer (#4), Shamamk (#5) Zone 2 (Central Erbil): Erbil (#6), Khabat (#7), Bnaslawa (#8), Koya (#9), and Taqtaq (#10).

**FIGURE A2:**
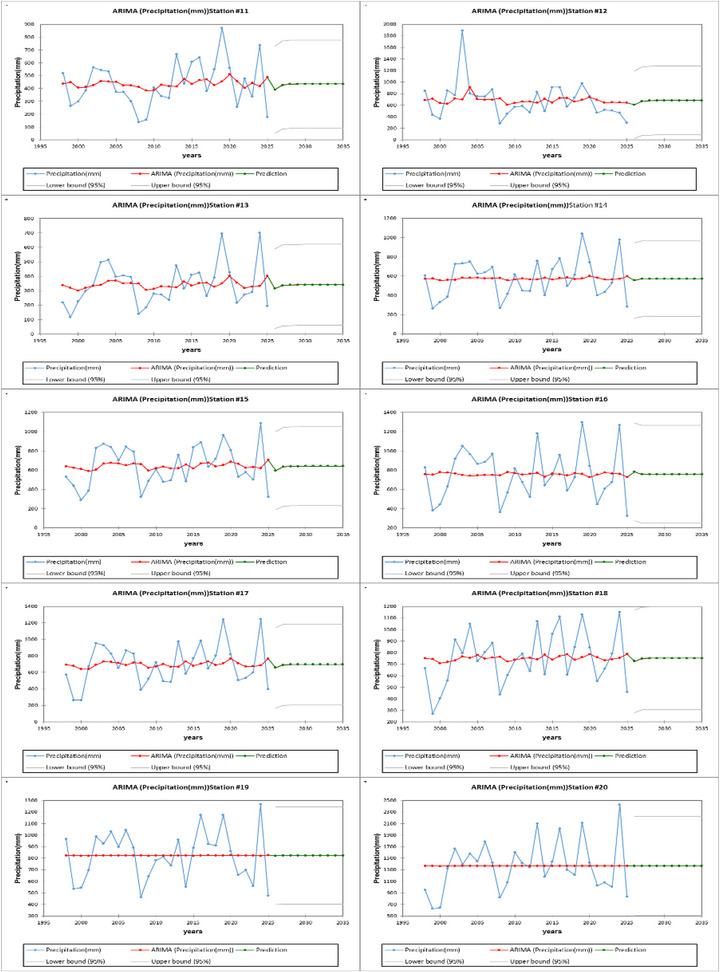
Historical Trends and ARIMA Forecasts of Annual Precipitation (mm) for Meteorological Stations Across Three Zones in Erbil Governorate (1998–2035): Zone 2 Taqtaq (#10), Bastora (#11) Zone 3 (Northern Erbil): Barzewa (#12), Harir (#13), Soran (#14), Shaqlawa (#15), Khalifan (#16), Choman (#17), Sidakan (#18), Rwanduz (#19), Mergasur (#20).

Over the 28 years, the three zones display a clear north–south gradient in both precipitation levels and stability (Figure 15; Figures A1, and A2). Zone 1, the Southern lowlands (Qushtapa, Makhmoor, Dibaga, Gwer, Shamamk), receives annual precipitation ranging from 95 to 645 mm, with an average of 272.7 mm and a standard deviation of 124.7 mm. The ARIMA (1,0,0) model accurately fits this highly variable semi‐arid series (RMSE = 117.6 mm; MAPE = 37.8%). The constant term (269.4 mm; 95% CI: 209.8–329.0) matches the sample mean, while the autoregressive component (AR (1) = 0.279) is weak and not statistically significant, indicating little persistence from year to year. Variability at the station level is high, with many stations exhibiting %CV over 40, consistent with the unstable precipitation patterns in the plains south of the foothills. This pattern aligns with regional climate, where the Zagros orography blocks or deflects moist westerlies inland, resulting in drier and more variable interior lowlands. Using %CV to summarize precipitation variability is a standard approach in hydroclimate analysis. Given the large prediction intervals and moderate accuracy typical for precipitation time series, ARIMA remains useful for short‐term planning but should be complemented with water harvesting and supplemental irrigation strategies [60, 61, 62].

Zone 2 ‐ Central transitional belt (Erbil, Khabat, Bnaslawa, Koya, Taqtaq, Bastora) has precipitation levels ranging from 159 to 778 mm, with a mean of 383.7 mm and a standard deviation of 140.5 mm. The ARIMA (1,0,0) model performs slightly better than Zone 1, with an RMSE of 136.8 mm and a MAPE of 33.9%, indicating more stable conditions in the foothill transition. The constant value (382.6 mm; 95% CI: 324.5–440.6) reflects the average; the AR (1) = 0.132 is again weak (since the CI includes 0), suggesting limited inter‐annual persistence. Spatially, Zone 2 shows intermediate coefficients of variation (∼34%–42%), consistent with a climatic gradient from semi‐arid south to the orographically enhanced north. The increasing moisture from south to north, linked to the Zagros barrier and upslope lifting, explains both higher totals and somewhat lower variability compared to Zone 1. Forecast uncertainty remains significant; although ARIMA is commonly used and accepted for hydrologic time‐series forecasting, accuracy metrics like MAPE have known limitations. Future comparisons across series should ideally include scale‐free measures such as MASE [60].

Zone 3 and Zone 2 ‐ Central transitional belt (Erbil, Khabat, Bnaslawa, Koya, Taqtaq, Bastora) have precipitation levels ranging from 159 to 778 mm, with an average of 383.7 mm and a standard deviation of 140.5 mm. The ARIMA (1,0,0) model performs slightly better than in Zone 1, with an RMSE of 136.8 mm and a MAPE of 33.9%, indicating more stable conditions in the foothill transition. The constant value (382.6 mm; 95% CI: 324.5–440.6) reflects the mean; the AR (1) = 0.132 is again weak (as the CI includes 0), suggesting limited inter‐annual persistence. Spatially, Zone 2 shows intermediate coefficients of variation (∼34%–42%), aligned with a climatic gradient from semi‐arid south to the orographically enriched north. The increasing moisture from south to north, influenced by the Zagros barrier and upslope lifting, accounts for both higher totals and somewhat lower variability compared to Zone 1. Forecast uncertainty remains high; although ARIMA models are commonly accepted for hydrologic time‐series forecasting, accuracy metrics like MAPE have known limitations. Future comparisons across series should ideally use scale‐free measures such as MASE.—Northern Mountains (Barzewa, Harir, Soran, Shaqlawa, Khalifan, Choman, Sidakan, Rwanduz, Mergasur). This zone is the wettest and relatively the most stable: 407–1,223 mm, mean = 778.2 mm, SD = 235.6 mm. Despite the larger absolute spread, ARIMA (1,0,0) shows the best relative accuracy (RMSE = 231.1 mm; MAPE = 28.8%), with a constant value of 777.4 mm (95% CI: 687.2–867.6) and negligible persistence (AR (1) = 0.053; CI includes 0). Station CVs are lowest (≈26%–35%), reflecting orographic enhancement by the Zagros, which produces higher and more reliable precipitation through uplift of Mediterranean/Persian Gulf air masses. This northward increase in totals, along with lower CV, is well documented for the Zagros and neighboring Kurdistan Region topography [60]. Practically, Zone 3 provides the most consistent conditions for rain‐fed agriculture, forestry, and water harvesting, although fluctuations from year to year still require soil and runoff management. Together, the zones show a north–south gradient in both amount and predictability: Zone 1 (dry, high CV, least predictable), Zone 2 (moderate), and Zone 3 (wet, lowest CV, most predictable).

This gradient aligns with Zagros orography and the established use of CV for variability assessment and ARIMA for short‐term hydrologic forecasting. When reporting forecast skill, remember that MAPE can be misleading for intermittent or low‐denominator values; it is best to use it alongside scale‐free metrics (e.g., MASE) in future analyses [61, 62].

## 4 Discussion

### 4.1 The Precipitation Map Interpolation

Precipitation map interpolation is vital for understanding how precipitation varies across space and time, which is crucial for climate research, water resource management, and disaster preparedness. The results from this study, based on data from 20 meteorological stations over 28 years, reveal notable fluctuations in precipitation across Erbil. Similar research in arid and semi‐arid regions has shown comparable declining patterns in precipitation, especially in response to climate change and regional atmospheric variations [63, 64, 65]. The precipitation trends in Iraq show increasing drought severity over recent decades, aligning with the falling precipitation observed in Erbil's southern and central areas [66]. Another study confirmed that northern, mountainous regions tend to receive more precipitation due to orographic lifting, consistent with the results showing higher precipitation in Mergasur [67]. The impact of data gaps, particularly during extreme weather years, presents another limitation, potentially underestimating short‐term fluctuations in precipitation. Future research should focus on integrating remote sensing data and satellite‐based precipitation estimates to enhance spatial coverage and improve interpolation accuracy. Studies like those [68] have demonstrated the benefits of incorporating satellite‐derived precipitation data with ground observations for better hydrological modeling. The findings have important implications for climate adaptation strategies, especially in water resource management, agriculture, and urban planning. A decrease in rainfall in southern Erbil has increased the risk of water shortages, affecting the cultivated land that depends on precipitation [69, 70]. To address this issue, policymakers should focus on developing sustainable water management strategies, including improved irrigation techniques and rainwater harvesting systems, to mitigate the effects of declining precipitation [71].

### 4.2 Crop Area Maps

These findings emphasize a significant mismatch between climatic potential and agricultural land use, with significant implications for managing agricultural droughts. Specifically, relying on semi‐arid and dry zones for farming highlights these regions' vulnerability to water shortages and prolonged droughts. Effective planning and resource allocation are essential to reduce drought impacts on Erbil's agriculture, especially in Zones 1 and 2, while also exploring sustainable use of the relatively underutilized wet Zone 3 for specific farming activities. The Crop Area Map illustrates the spatial distribution of cultivated lands, focusing on areas mainly used for winter crops like wheat and barley. These crops are known for their drought‐resistant qualities, making them suitable for regions with variable rainfall. In Erbil, wheat and barley are commonly cultivated in Zones 1 and 2, which feature dry and semi‐arid conditions, respectively. These zones receive less rainfall but still enough to support these hardy crops. Zone 3, identified as a wet zone, receives significantly more rainfall but has limited cultivated areas, primarily due to its rugged mountainous terrain that restricts large‐scale farming. The distribution of wheat and barley demonstrates an adaptation to climatic conditions, where rainfall levels in Zones 1 and 2, though modest, are sufficient to support these drought‐resistant crops. Meanwhile, the higher rainfall in Zone 3 remains underused due to topographical challenges rather than climatic unsuitability. Overall, these findings highlight a crucial mismatch between climatic potential and land use, with important implications for drought management. Relying on semi‐arid and dry zones for agriculture increases the vulnerability of these areas to water shortages and prolonged droughts. Strategic planning and resource distribution are essential to lessen drought effects on Erbil's agriculture, particularly in Zones 1 and 2, and to sustainably utilize the underused wet Zone 3 for targeted agricultural activities.

Several studies have explored the relationship between climate variability and agricultural land use in semi‐arid regions. For example [72], investigated the impact of drought on wheat and barley production in northern Iraq and found that erratic precipitation patterns significantly influenced yield variability. Similarly, studies by Saeed et al. [73] highlight that water resource management is crucial for sustaining agriculture in arid and semi‐arid regions. The findings in this study support this, showing that despite moderate rainfall in Zones 1 and 2, agricultural activities remain vulnerable to climate variability. However, our results also indicate that Zone 3, which receives much higher rainfall, is still underused due to topographical challenges. This aligns with the work of Jaradat, AA [74], who noted that terrain limitations often hinder agricultural expansion despite favorable climatic conditions. Additionally, recent studies have emphasized the role of climate‐smart agriculture in mitigating the adverse effects of drought [75]. Incorporating techniques such as conservation tillage and drought‐resistant crop varieties has been suggested as an adaptive strategy in arid regions [71]. Our findings further reinforce the need for such measures, particularly in Zones 1 and 2, where water scarcity remains a pressing concern.

While this study offers valuable insights into the spatial distribution of cultivated lands in Erbil, agricultural land use remains dynamic, and climate variability may affect cultivation patterns over more extended periods. A multi‐year analysis incorporating remote sensing and field surveys could provide a deeper understanding of long‐term trends and adaptation strategies. The findings of this study have several implications for sustainable agricultural planning in Erbil. First, policymakers should focus on drought mitigation strategies in Zones 1 and 2 through improved irrigation infrastructure, water conservation techniques, and the promotion of climate‐resilient crops. Second, Zone 3 offers an opportunity for alternative agricultural activities. While large‐scale farming may be impractical due to terrain constraints, small‐scale, high‐value agriculture such as orchards, vineyards, and agroforestry could be encouraged. Finally, integrating advanced technologies like precision agriculture and remote sensing can enhance the monitoring and management of agricultural land use.

### 4.3 Reference Evapotranspiration (ETo), Crop Evapotranspiration (ETc), and Actual Evapotranspiration (ETa)

The severity of drought across different agricultural zones has been assessed using a combination of the Standardized Precipitation Index (SPI), coefficient of variation results, and other weather indicators over three selected years categorized as dry, average, and wet years. The results highlight considerable variability in water availability for agriculture, which has been further analyzed through Tables A3, A4, A5, confirming the consistency of these factors. The detection of unexpected changes in reference evapotranspiration (ETo) showed significant variations in ETo measurements across various stations during different years. These sudden shifts can be linked to changes in climatic factors such as wind speed and relative humidity. Additionally, studies have suggested that changes in irrigation practices can influence ETo readings [76, 77]. The wet day metric indicates how often days with measurable precipitation occur. Higher values in wet years, as expected, indicate more frequent rainfall, which affects moisture availability and could influence the timing of agricultural activities [78].

Figure 7, 8, 9 and Table A3, A4, A5 provide a detailed assessment of various agricultural water management and climate‐related parameters, including precipitation (Prc.), reference evapotranspiration (ETo), crop evapotranspiration under standard conditions (ETc), actual evapotranspiration (ETa), the number of days suitable for crop growth (Crop Days), crop water deficit (Crop Deficit), drainage (Drain), and soil water content (Soil Water). These indicators differ significantly across diverse climate conditions, influencing moisture availability and the timing of agricultural activities [79]. In a wet year, March can see up to 186 mm of precipitation, whereas in a dry year, only 9 mm occurs during the same period. These variations significantly impact soil moisture, crop water availability, and overall agricultural productivity. Crop Deficit reflects the difference between (ETc) and (ETa), indicating water stress. For example, in June of a dry year, the crop deficit is notably high (277 mm m^−1^), highlighting significant water stress.

Drainage (Drain) refers to the water that moves past the root zone. Notable drainage in March of a wet year (59 mm/m) suggests possible over‐irrigation or excessive precipitation. Soil Water Content shows the moisture levels in the soil, fluctuating significantly. Moisture content tends to be higher in months with more rainfall and irrigation but drops notably during dry periods or when crops exhibit higher water usage. This analysis helps understand water movement under different weather conditions and can be crucial for developing effective water management strategies in agriculture, especially amid changing climate conditions conditions [78]. A critical finding from this study is the detection of unexpected changes in reference evapotranspiration (ETo) measurements across multiple stations during distinct years. These variations appear to be driven by climatic factors such as wind speed and relative humidity, which align with findings by Hameed [76]. Previous research has also suggested that modifications in irrigation practices influence ETo readings [80], which is an essential consideration for future water management strategies. Crop water deficit (Crop Deficit) is particularly concerning in dry years, as exemplified by a deficit of 277 mm/m in June, indicating severe water stress. This aligns with previous studies that highlight the increasing vulnerability of agriculture to climate‐induced droughts [79].

Future research should focus on incorporating high‐resolution remote sensing data and machine learning models to improve drought prediction and water management strategies. The integration of satellite‐based soil moisture measurements and real‐time climate monitoring can enhance the accuracy of drought assessments and early warning systems. Additionally, the development of adaptive irrigation scheduling models, tailored to specific crop water requirements under varying climatic conditions, could significantly improve water‐use efficiency.

### 4.4 Standardized Precipitation Index (SPI)

The spatiotemporal trends of the SPI for 20 meteorological stations in Erbil, reveal significant variations in drought severity across the region. This study confirms that drought conditions fluctuate over time. Over the past twenty‐four years, the frequency of extreme drought events has increased approximately three‐ to four‐fold compared to earlier decades, consistent with global and regional climate variability trends. These findings align with previous studies indicating that rising global temperatures and changing precipitation patterns contribute to increased drought frequency and severity [81]. The distribution patterns of SPI across Erbil's sub‐districts demonstrate spatial variability in drought severity from 1999 to 2021 (Figures 11 and Tables 5).

Several studies have reported similar findings for Erbil semi‐arid regions, where localized climatic factors and topographical influences contribute to diverse drought impacts [10, 82]. The analysis also highlights the presence of cyclic dry and wet spells, suggesting an oscillatory behavior in precipitation patterns. These cyclical drought patterns have been observed in other semi‐arid regions globally, such as the Mediterranean basin and parts of the Middle East [79].

The severity of drought conditions in Erbil has had profound implications for agricultural productivity and water resource management. During periods of severe drought, reductions in crop yields and groundwater depletion were reported, similar to findings in other drought‐prone regions [83]. The increasing frequency of severe drought events underscores the need for adaptive water management strategies, particularly in agricultural zones reliant on seasonal precipitation. Moreover, the comparison of SPI‐derived findings with those from the (CV) index (Figure 12, 13, 14) supports the reliability of the drought classification methodology used in this study.

The concurrence of these indices further reinforces the robustness of SPI as an effective tool for drought assessment, in agreement with prior research [56]. During specific periods, such as 1999–2000, 2008–2009, 2020–2021, and 2024–2025, droughts became extremely severe, whereas other times, like 2012, only experienced moderate cases. This suggests that, regardless of intensity, drought can occur even when average precipitation appears normal for hydrological or vegetative conditions. The frequency of severe to extreme droughts increased three to four times compared to earlier decades within Erbil's borders. Additionally, this research shows that intermittent events had significant impacts on agricultural practices and water supply issues. Spatiotemporal analyses revealed variation among different sub‐districts in the severity of prolonged episodes lasting several years. The trend alternated between drier and wetter cycles, but no clear overall trend was observed across all studied years (Figure 12, 13, 14).

According to [84], A negative value of the Standardized Precipitation Index (SPI) indicates drought, while positive values signify its absence. Throughout the studied years, approximately 57% fall into the near‐normal drought category with an SPI range between −1 and +1 (Table 3, 4). The findings of this study have important implications for regional water resource management, agricultural planning, and climate resilience strategies. Given the rising frequency of extreme droughts, proactive policies should be developed to improve water conservation and enhance drought preparedness. Implementing early warning systems based on SPI and other hydroclimatic indicators could help decision‐makers mitigate drought‐related risks.

### 4.5 Coefficient Variation of Annual and Monthly Precipitation

The (CV) of precipitation is a key metric for understanding how much precipitation varies from its average. It offers useful insights into the challenges and opportunities for agricultural planning and water resource management. Higher CV values, especially in the southern parts of Erbil, show significant fluctuations in precipitation, which can complicate agricultural practices and water management strategies. These areas are more vulnerable to unpredictable water supplies, making rain‐fed agriculture particularly at risk during dry periods and increasing the likelihood of drought [85]. On the contrary, the northern areas of Erbil, including Mergasur, have lower CV values because of their higher elevation and consistent precipitation patterns. This stability contributes to more reliable agricultural yields and decreases vulnerability to drought, emphasizing the significance of geographic and climatic elements in influencing local agricultural methods [86].

The spatial distribution of crops, as shown in crop area maps, does not align with these precipitation variability trends. Crops like wheat and barley, which require a consistent water supply, are mainly grown in areas with high CV values. This suggests strategic farming choices that overlook environmental factor conditions [87]. Understanding the link between the CV and crop distribution is crucial for enhancing agricultural productivity and sustainability. Precipitation variability, represented by the CV, greatly affects the assessment of drought and wet periods through the (SPI). Regions with higher CV values are more prone to frequent shifts between drought and wet conditions, resulting in greater fluctuations in SPI readings [88]. Fluctuations in precipitation levels make it challenging to assess the risk of drought, requiring adaptive management strategies to mitigate the impacts of this variability. Moreover, measures of evapotranspiration (ETo, ETc, ETa) are directly affected by the variability in precipitation. In areas or during years with a high (CV), there can be significant differences between potential (ETo) and actual (ETa) evapotranspiration, indicating either water stress or inefficiency in water utilization [89]. This highlights the need for precise water management practices to address the challenges posed by variable precipitation.

Understanding the (CV) of precipitation, along with other environmental data, is essential for developing effective strategies to reduce risks in agriculture. Farmers and agricultural planners can use this information to make informed decisions about crop selection, irrigation timing, and investment in water conservation methods. In areas with a high (CV), where precipitation shows significant variability, it is crucial to build strong infrastructure for water retention and irrigation to effectively manage the heightened risk of drought. Conversely, in regions with a low (CV) and consistent precipitation, the emphasis can be on improving crop yields and overall agricultural productivity [90, 91]. Regional agricultural policies and strategies can be tailored based on the environmental variability depicted by CV, SPI, and evapotranspiration data. This allows for more precise and efficient interventions aimed at improving agricultural productivity and sustainability [92].

Finally, the implications of climate change on precipitation patterns and drought risk should be examined further. As global temperatures continue to rise, the frequency and intensity of extreme weather events, including droughts, are expected to increase [93].

## 5 Conclusion

This study investigates the spatio‐temporal patterns of precipitation and drought in Erbil Districts from 1998 to 2025, highlighting the interaction between climate variability and agricultural practices.

1. Key findings show a recurring pattern of severe drought years alternating with periods of normal to above‐average rainfall, emphasizing the vulnerability of Erbil's agricultural systems, especially in the semi‐arid and dry zones (Zones 1 and 2), to water shortages and prolonged droughts.
2. The study underscores the importance of understanding precipitation variability and its effects on water resource management, agricultural planning, and drought mitigation. Challenges caused by fluctuating rainfall in southern Erbil and more stable patterns in the north highlight the need for resilient farming practices and may limit rain‐fed agriculture. To address these issues, strategic actions such as expanding water storage infrastructure, optimizing irrigation methods, and promoting drought‐resistant crops are essential.
3. The study underscores the necessity of proactive planning and climate adaptation strategies to cope with the cyclical nature of wet and dry periods, as well as the rising frequency of extreme droughts due to climate change.
4. While offering valuable insights, the study also recognizes limitations like reliance on historical meteorological data and the omission of socioeconomic factors. Future research should include higher‐resolution spatial analyses, long‐term climate trend examination, and interdisciplinary approaches to better understand drought impacts and adaptive capacities.
5. The findings highlight the significance of holistic strategies in managing precipitation variability, soil water balance, and drought resilience for sustainable agriculture in Erbil. These insights can help policymakers develop better land and water management policies in the Kurdistan Region of Iraq (KRI), supporting the achievement of SDG 2 endorsed by the UN and aligning with Nuffic program goals for agricultural planners and regional authorities.

## Author Contributions

Conceptualization, P.A, H.A.A.G, S.H, M.R, and K.M; methodology, P.A, H.A.A.G, S.H, M.R, F.A, K.H and K.M.; resources, H.G and P.A.; data curation, H.G and P.A.; formal analysis, and H.G.; writing—original draft preparation, H.A.A.G, S.H, M.R, F.A, K.H and K.M; writing—review and editing, H.A.A.G, S.H, M.R, and K.M; visualization, and H.A.A.G.; supervision, D.R.K. and E.A.; Scientific editing, and H.A.A.G. K.M; funding acquisition, F.A, K.H, M.R, and K.M All authors have read and agreed to the published version of the manuscript.

## Funding

This study was supported by the Orange Knowledge Programme of Nuffic, funded by the Ministry of Foreign Affairs of the Netherlands (OKP‐IRA‐104278). This research was partially funded by Wageningen university & research, and Salahaddin University‐Erbil.

## Conflicts of Interest

The authors declare no conflicts of interest.

## Data Availability

The data that support the findings of this study are available from the corresponding author upon reasonable request.

## APPENDIX A

### A.1

**TABLE A1:** The mean and coefficient of variation (CV%) for Annual precipitation over a 28‐year span for thirteen selected meteorological stations across the Erbil Governorate.

Longitude	Latitude	Station no.	Mean(mm)	S.dv	CV%	Max precipitation(mm)	Min precipitation(mm)
**44.028**	**36.001**	**#1**	308.5	138.6	44.9	681.5	106.1
**43.583**	**35.783**	**#2**	239.9	105.2	43.9	530.3	88.5
**43.805**	**35.873**	**#3**	263.4	121.2	46.0	663.9	94.0
**43.481**	**36.045**	**#4**	255.6	132.2	51.7	601.6	93.0
**43.847**	**36.040**	**#5**	296.1	141.6	47.8	746.4	91.0
**44.009**	**36.191**	**#6**	333.8	132.7	39.7	645.6	114.2
**43.674**	**36.273**	**#7**	312.3	123.4	39.5	733.0	125.7
**44.140**	**36.154**	**#8**	342.5	143.1	41.8	700.0	118.0
**44.648**	**36.099**	**#9**	494.6	184.5	37.3	1047.6	204.9
**44.586**	**35.887**	**#10**	383.0	130.7	34.1	677.6	151.3
**44.160**	**36.339**	**#11**	436.1	174.8	40.1	870.4	139.7
**44.633**	**36.627**	**#12**	710.8	307.9	43.3	1889.0	284.2
**44.365**	**36.551**	**#13**	573.8	199.7	34.8	1042.1	264.5
**44.561**	**36.638**	**#14**	643.5	209.0	32.5	1085.0	290.5
**43.985**	**36.209**	**#15**	756.6	259.1	34.2	1295.5	326.4
**44.404**	**36.599**	**#16**	698.4	250.3	35.8	1243.8	263.6
**44.889**	**36.637**	**#17**	752.9	226.7	30.1	1152.5	271.3
**44.671**	**36.797**	**#18**	823.1	215.1	26.1	1265.8	463.7
**44.525**	**36.612**	**#19**	706.4	209.1	29.6	1123.3	342.4
**44.306**	**36.838**	**#20**	1365.4	436.6	32.0	2430.0	624.7

**TABLE A2:** The coefficient of variation (CV%) for monthly precipitation over a 24‐year span for thirteen selected meteorological stations across the Erbil Governorate, namely: Erbil = 1, Qushtapa = 2, Shaqlawa = 3, Mergasur = 4, Sidakan = 5, Maxmur = 6, Gwer = 7, Xabat = 8, Xalifan = 9, Soran = 10, Choman = 11, Rwandz = 12 and Bastora = 13.

—	Erbil	Qushtapa	Shaqlawa	Mergasur	Sidakan	Maxmur	Gwer	Xabat	Xalifan	Soran	Choman	Rwandz	Bastora
**January**	0.56	0.57	0.65	0.64	0.47	0.61	0.57	130.70	0.50	0.57	0.63	0.51	0.65
February	0.70	0.79	0.60	0.57	0.49	1.11	0.59	134.60	0.61	0.66	0.60	0.68	0.55
March	0.71	0.85	0.63	0.57	0.50	1.00	0.92	217.50	0.53	0.51	0.43	0.47	0.71
April	0.71	0.81	0.68	0.70	0.65	0.77	0.86	122.40	0.71	0.63	0.62	0.65	0.73
May	1.15	1.15	0.89	0.73	0.88	1.07	1.12	41.60	1.31	0.92	0.74	1.04	1.11
October	1.19	1.28	1.17	1.28	1.18	1.13	1.02	65.90	1.25	1.30	1.17	1.19	1.33
November	0.99	1.37	0.87	0.66	0.66	1.05	0.98	158.40	0.70	0.79	0.70	0.71	0.87
December	0.69	0.77	0.73	0.66	0.55	0.63	0.65	200.10	0.70	0.65	0.69	0.56	0.70

**TABLE A3:** Soil Water Balance during a Wet year, Dry year, and a Normal Year in (Zone‐1).

—	Prc.	Wet days	ETo	ETc	ETa	Crop days	ETc‐crop	Crop deficit	Drain	Soil water
Month	(mm/m)	days	(mm/d)	(mm/m)	(mm/m)	days	(mm/m)	(mm/m)	(mm/m)	(mm)
Wet year										
Jan	92	19	46	25	25	31	25	0	0	187
Feb	62	19	53	49	49	28	49	0	0	200
Mar	186	22	80	76	76	31	76	0	59	240
Apr	90	22	116	110	110	30	110	0	2	191
May	16	11	219	208	139	31	208	69	0	58
Jun	1	3	299	284	23	30	284	261	0	33
Jul	0	0	299	280	2	31	280	278	0	31
Aug	0	5	279	189	0	13	111	111	0	30
Sep	0	0	201	101	0	0	0	0	0	30
Oct	42	13	141	71	6	0	0	0	0	67
Nov	29	6	86	43	32	0	0	0	0	63
Dec	79	18	45	22	22	0	0	0	0	120
Dry year										
Jan	46	17	50	27	20	31	27	7	0	48
Feb	31	14	64	59	42	28	59	18	0	38
Mar	9	17	129	122	31	31	122	91	0	15
Apr	12	11	182	173	20	30	173	153	0	8
May	1	8	273	259	5	31	259	254	0	4
Jun	0	2	292	278	1	30	278	277	0	4
Jul	1	2	317	298	1	31	298	297	0	4
Aug	0	1	299	205	0	13	124	124	0	4
Sep	0	2	204	102	0	0	0	0	0	4
Oct	7	10	146	73	1	0	0	0	0	10
Nov	5	7	79	40	8	0	0	0	0	7
Dec	22	7	52	26	13	0	0	0	0	15
Normal Year										
Jan	87	24	39	22	22	31	22	0	0	135
Feb	79	19	53	49	49	28	49	0	0	165
Mar	7	10	120	114	110	31	114	5	0	61
Apr	57	16	160	152	71	30	152	81	0	47
May	12	16	220	209	45	31	209	163	0	14
Jun	0	0	287	272	6	30	272	267	0	8
Jul	0	2	304	285	1	31	285	285	0	7
Aug	0	0	282	192	0	13	113	113	0	7
Sep	0	0	219	110	0	0	0	0	0	7
Oct	2	7	152	76	1	0	0	0	0	8
Nov	64	21	65	32	21	0	0	0	0	52
Dec	30	9	39	19	19	0	0	0	0	63

**TABLE A4:** Soil Water Balance during a Wet year, dry year and Normal Year in (Zone 2).

Month	Prc.	Wet days	ETo	ETc	ETa	Crop days	ETc‐crop	Crop deficit	Drain	Soil water
—	(mm/m)	days	(mm/d)	(mm/m)	(mm/m)	days	(mm/m)	(mm/m)	(mm/m)	(mm)
Wet Year
Jan	158	20	38	21	21	31	21	0	45	240
Feb	114	20	46	43	43	28	43	0	44	240
Mar	255	21	68	65	65	31	65	0	157	240
Apr	127	24	109	103	103	30	103	0	35	200
May	20	11	188	179	138	31	179	41	0	67
Jun	1	4	234	223	28	30	223	195	0	35
Jul	0	0	233	219	3	31	219	216	0	31
Aug	0	4	221	152	0	13	92	91	0	31
Sep	0	0	161	81	0	0	0	0	0	31
Oct	29	14	119	59	6	0	0	0	0	54
Nov	45	6	68	34	22	0	0	0	0	78
Dec	94	21	38	19	19	0	0	0	0	152
Dry year										
Jan	55	19	43	23	23	31	23	0	0	78
Feb	91	13	52	48	48	28	48	0	0	121
Mar	23	17	101	96	90	31	96	6	0	54
Apr	26	14	145	138	60	30	138	77	0	20
May	2	10	224	213	15	31	213	199	0	7
Jun	0	3	238	226	2	30	226	225	0	6
Jul	3	4	258	242	2	31	242	240	0	6
Aug	0	1	233	157	1	13	91	90	0	6
Sep	0	1	165	82	0	0	0	0	0	6
Oct	10	12	119	59	4	0	0	0	0	12
Nov	10	11	63	31	10	0	0	0	0	13
Dec	43	7	45	23	15	0	0	0	0	41
Normal year										
Jan	80	19	33	18	18	31	18	0	0	217
Feb	135	23	40	38	38	28	38	0	51	237
Mar	96	12	78	74	74	31	74	0	5	229
Apr	61	19	127	121	121	30	121	0	13	135
May	6	14	196	186	87	31	186	99	0	46
Jun	0	0	232	221	11	30	221	209	0	32
Jul	0	0	240	225	1	31	225	224	0	30
Aug	0	1	237	162	0	13	96	96	0	30
Sep	0	0	161	80	0	0	0	0	0	30
Oct	2	8	106	53	1	0	0	0	0	32
Nov	50	12	55	28	15	0	0	0	0	66
Dec	109	15	31	16	16	0	0	0	0	159

**TABLE A5:** Soil Water Balance during a Wet year, dry year and Normal Year in (Zone 3).

Month	Prc.	Wet days	ETo	ETc	ETa	Crop days	ETc‐crop	Crop deficit	Drain	Soil water
—	(mm/m)	days	(mm/d)	(mm/m)	(mm/m)	days	(mm/m)	(mm/m)	(mm/m)	(mm)
Wet year										
Jan	125	20	29	16	16	31	16	0	86	233
Feb	236	20	35	32	32	28	32	0	171	239
Mar	103	23	77	73	73	31	73	0	10	230
Apr	107	26	101	96	96	30	96	0	0	225
May	166	26	131	125	125	31	125	0	75	165
Jun	4	9	176	167	104	30	167	63	0	53
Jul	0	0	199	187	16	31	187	171	0	34
Aug	1	3	182	123	2	13	71	70	0	32
Sep	2	5	136	68	2	0	0	0	0	33
Oct	164	22	83	41	12	0	0	0	0	182
Nov	130	24	35	17	17	0	0	0	32	240
Dec	300	24	23	11	11	0	0	0	255	240
Dry year										
Jan	78	21	29	16	16	31	16	0	0	186
Feb	153	15	37	34	34	28	34	0	67	220
Mar	82	20	69	66	66	31	66	0	0	220
Apr	57	20	108	102	102	30	102	0	3	150
May	8	14	165	157	98	31	157	59	0	52
Jun	1	7	188	179	17	30	179	162	0	34
Jul	4	5	203	190	5	31	190	186	0	32
Aug	0	9	186	126	1	13	74	73	0	31
Sep	1	2	134	67	0	0	0	0	0	31
Oct	28	14	87	43	10	0	0	0	0	48
Nov	37	15	42	21	19	0	0	0	0	67
Dec	72	8	30	15	15	0	0	0	0	124
Normal year										
Jan	130	26	24	13	13	31	13	0	73	240
Feb	143	18	34	32	32	28	32	0	100	223
Mar	194	21	58	55	55	31	55	0	95	238
Apr	52	23	103	98	98	30	98	0	0	171
May	46	22	148	141	130	31	141	10	0	80
Jun	3	7	184	174	41	30	174	133	0	39
Jul	0	4	200	188	7	31	188	181	0	31
Aug	1	5	186	126	1	13	74	73	0	31
Sep	4	6	136	68	4	0	0	0	0	31
Oct	85	18	83	42	10	0	0	0	0	106
Nov	83	17	39	20	20	0	0	0	0	169
Dec	204	18	25	13	13	0	0	0	106	228
